# Supplementary figures and images for: Opioid overdose and naloxone administration knowledge and perceived competency in a probability sample of Indiana urban communities with large Black populations
Source: PLoS One. 2025 Jul 15;20(7):e0328444. doi: 10.1371/journal.pone.0328444 (PMC12262839; doi:10.1371/journal.pone.0328444)

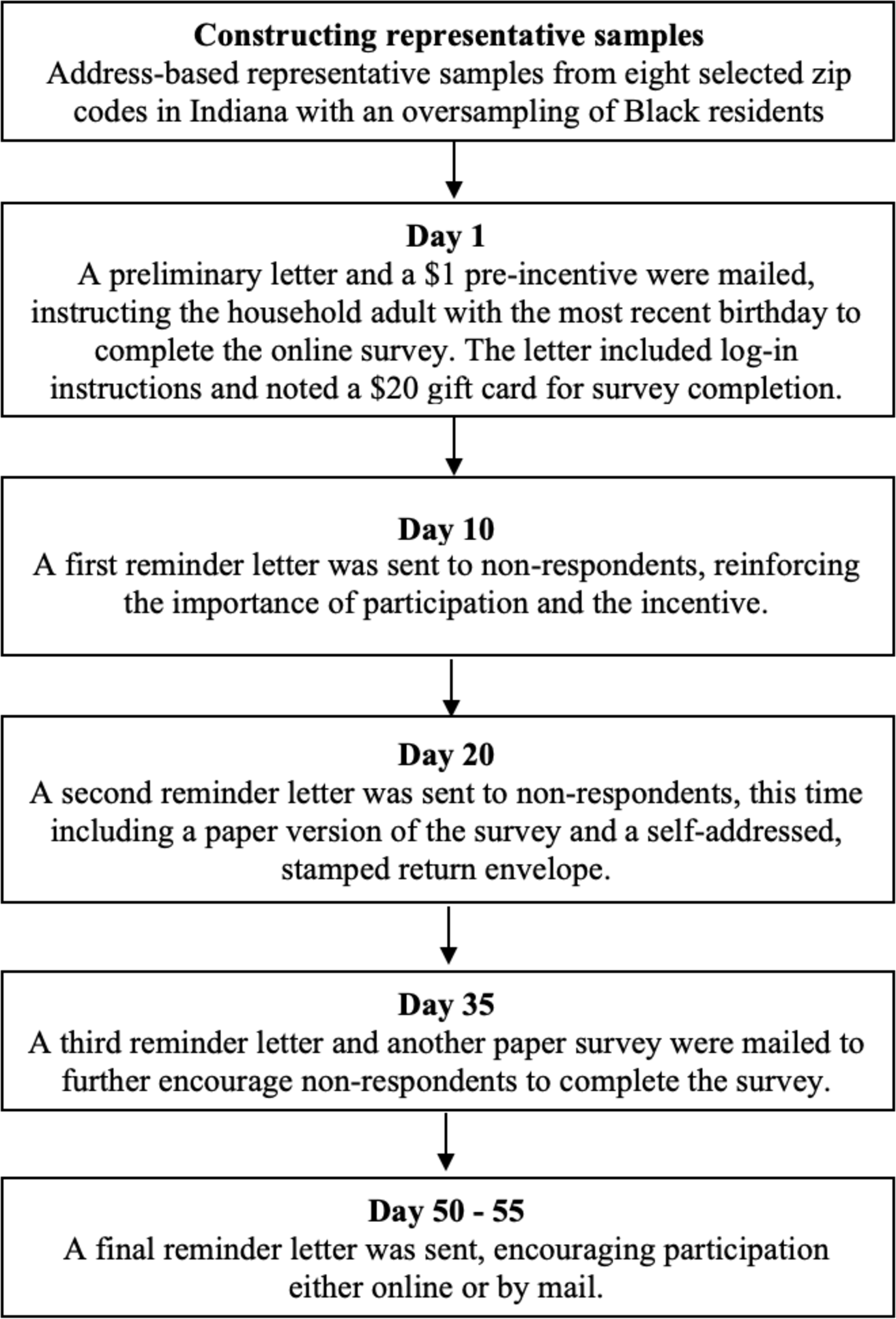

Supplement: S1 Fig — (TIF) [file pone.0328444.s001.tif]
