# Supplementary material for: Opioid overdose and naloxone administration knowledge and perceived competency in a probability sample of Indiana urban communities with large Black populations
Source: PLoS One. 2025 Jul 15;20(7):e0328444. doi: 10.1371/journal.pone.0328444 (PMC12262839; doi:10.1371/journal.pone.0328444)
Supplement: S1 Table — Frequencies may not sum to the total and proportions may not sum to 100% due to missing observations. (DOCX) [file pone.0328444.s002.docx]

**S1 Table. Demographic characteristics of survey respondents: March-May 2023 (N = 772)**

| Variable | n, unweighted | %, weighted |
| --- | --- | --- |
| *Sociodemographic variables* |  |  |
| Race |  |  |
| White | 359 | 41.6 |
| Black | 347 | 48.0 |
| Asian | 22 | 4.4 |
| American Indian or Alaska Native | 21 | 3.7 |
| Native Hawaiian or Pacific Islander | 6 | 1.8 |
| Ethnicity |  |  |
| Non-Latine | 697 | 90.1 |
| Latine | 65 | 9.5 |
| Age group |  |  |
| 18-24 years | 76 | 15.6 |
| 25-34 years | 128 | 18.7 |
| 35-44 years | 133 | 16.9 |
| 45-54 years | 102 | 12.6 |
| 55-64 years | 121 | 16.5 |
| 65-74 years | 133 | 12.3 |
| 75 years or older | 74 | 7.3 |
| Biological sex at birth |  |  |
| Women | 506 | 58.1 |
| Men | 261 | 41.9 |
| Educational attainment |  |  |
| Some college or higher | 587 | 57.7 |
| High school /GED or less | 182 | 42.3 |
| Household income |  |  |
| Upper, more than $100,000 | 154 | 20.5 |
| Middle, $35,000 - $99,999 | 335 | 38.8 |
| Lower, less than $35,000 | 271 | 40.7 |
| Length of time lived in the communities |  |  |
| More than three years | 549 | 69.8 |
| One year to three years | 164 | 23.4 |
| Less than a year | 55 | 6.8 |
| Opioid overdose history^a^ |  |  |
| No | 595 | 76.4 |
| Yes | 173 | 23.6 |
| Region |  |  |
| Indianapolis | 423 | 45.2 |
| Other | 349 | 54.8 |
