# Supplementary material for: Opioid overdose and naloxone administration knowledge and perceived competency in a probability sample of Indiana urban communities with large Black populations
Source: PLoS One. 2025 Jul 15;20(7):e0328444. doi: 10.1371/journal.pone.0328444 (PMC12262839; doi:10.1371/journal.pone.0328444)
Supplement: S2 Table — Frequencies may not sum to the total due to missing observations. Corresponding p-values for “a” were calculated via weighted adjusted Chi-square tests. Corresponding p-values for “b” were calculated via weighted design-based F-test. (DOCX) [file pone.0328444.s003.docx]

S3 Table. Weighted frequencies and percentages for knowledge on opioid overdose and naloxone administration by race: March-May, 2023 (N = 772)

|  | White  (n = 359) | Black  (n = 347) | Other  (n = 60) |  |
| --- | --- | --- | --- | --- |
| *Weighted Percentage of Correct Response - True or False Statement (6 Questions)* | n (%) | | | *p*-value^b^ |
| Naloxone is effective in reversing effects of cocaine overdose. (False) | 157 (43.4) | 96 (30.3) | 24 (43.2) | 0.055 |
| All naloxone products are effective in reversing opioid overdose, including fentanyl-involved opioid overdoses. (True) | 219 (58.6) | 216 (63.8) | 31 (53.2) | 0.446 |
| Naloxone should NOT be used for pregnant women in life-threatening opioid overdose circumstances. (False) | 231 (58.5) | 147 (43.0) | 21 (36.6) | 0.012 |
| Because naloxone’s effects don’t last long, overdose symptoms may come back. (True) | 230 (68.7) | 192 (56.7) | 36 (54.9) | 0.082 |
| Naloxone can provoke withdrawal symptoms like fever, irritability, rapid heart rate, sweating, nausea, and vomiting. (True) | 278 (78.0) | 250 (74.1) | 44 (85.4) | 0.253 |
| If the person overdosing does not respond within 2 to 3 minutes after administering a dose of naloxone, a second dose of naloxone can be administered. (True) | 255 (70.6) | 216 (57.4) | 38 (64.8) | 0.070 |
|  |  |  |  |  |
| *Weighted Percentage of Correct Response - Multiple Choices Questions (4 Questions)* | n (%) | | | *p*-value^b^ |
| Which of the following is NOT a sign or symptom of opioid overdose?   1. Slow or shallow breathing 2. Having blood-shot eyes (Correct) 3. Unconsciousness 4. Very small pupils 5. Fingernails or lips turning blue/purple | 229 (66.8) | 190 (52.6) | 26 (39.4) | 0.005 |
| Which step is NOT recommended when someone has overdosed on opioids?   1. Make the person vomit the drugs that may have been swallowed (Correct) 2. Call 911 for help 3. Monitor what is happening to the person until an ambulance arrives 4. Be sure the person’s airway is clear | 302 (86.3) | 255 (72.3) | 51 (88.4) | 0.007 |
| How should you administer naloxone?   1. Spray through the nose 2. Inject directly into the muscle 3. Inject directly under the skin 4. All of them are correct routes of administration (Correct) | 202 (53.2) | 215 (62.5) | 29 (55.6) | 0.282 |
| Which is a CORRECT statement?   1. There is no need to call for an ambulance if I know how to manage an overdose. 2. After recovering from an opioid overdose, the person must not take any opioid, but it is okay for them to drink alcohol or take sleeping tablets. 3. You should not give naloxone more than once to people who suffer from a fentanyl-involved overdose and taking breaths that are slower and shallower than normal. 4. Someone can overdose again even after having received naloxone. (Correct) | 358 (81.2) | 340 (66.6) | 58 (54.3) | 0.002 |
| *Weighted Mean of Each Perceived Competency to Manage Opioid Overdose Item* | Mean (SD) | | | *p*-value^a^ |
| 1. I would be able to respond effectively with an overdose | 3.02 (1.35) | 2.74 (1.41) | 3.21 (1.50) | 0.110 |
| 2. I am confident that I can administer naloxone to someone who has overdosed | 3.00 (1.47) | 3.18 (1.41) | 3.13 (1.47) | 0.579 |
| 3. If someone overdoses, I would know what to do to help them | 3.20 (1.28) | 3.01 (1.36) | 3.36 (1.47) | 0.282 |
| 4. I know very little about how to help someone who has overdosed | 2.82 (1.38) | 2.64 (1.44) | 2.65 (1.49) | 0.530 |
| 5. I would be afraid of doing something wrong in an overdose situation | 2.38 (1.33) | 2.41 (1.36) | 2.29 (1.34) | 0.915 |
| 6. I would be afraid of giving naloxone in case the person becomes aggressive afterwards | 3.14 (1.35) | 2.83 (1.34) | 3.00 (1.11) | 0.153 |
| 7. If I tried to help someone who has overdosed, I might accidentally hurt them | 3.09 (1.18) | 2.97 (1.29) | 3.11 (1.34) | 0.701 |
