# Supplementary material for: Opioid overdose and naloxone administration knowledge and perceived competency in a probability sample of Indiana urban communities with large Black populations
Source: PLoS One. 2025 Jul 15;20(7):e0328444. doi: 10.1371/journal.pone.0328444 (PMC12262839; doi:10.1371/journal.pone.0328444)
Supplement: S3 Table — FPL = Federal Poverty Level. HS = high school. The “a” refers to the number of individuals with income below the 200 percent of federal poverty level. The “b” refers to the proportion of individuals with high school or higher educational attainment among those with 25 years or older. The “c” refers to the population size of each zip code. (DOCX) [file pone.0328444.s004.docx]

S4 Table. Zip-code level predictors: 2022 American Community Survey 5-year estimates

| Zip code | Black, % | ^a^FPL<200% | Median household income, $ | ^b^HS or higher, % | Employment rate, % | Population^c^ |
| --- | --- | --- | --- | --- | --- | --- |
| 46202 | 20.8 | 7,238 | 59,578 | 90.4 | 65.7 | 20,688 |
| 46205 | 36.7 | 8,788 | 63,161 | 92.7 | 71.0 | 28,327 |
| 46208 | 45.5 | 9,911 | 46,693 | 91.5 | 57.6 | 22,729 |
| 46218 | 68.6 | 16,501 | 32,772 | 76.4 | 47.2 | 28,951 |
| 46408 | 45.8 | 7,743 | 46,690 | 85.2 | 47.4 | 15,368 |
| 46410 | 47.2 | 13,019 | 60,432 | 92.1 | 56.5 | 39,121 |
| 46628 | 30.5 | 11,099 | 52,882 | 89.3 | 58.1 | 27,344 |
| 46806 | 41.3 | 14,562 | 39,615 | 75.4 | 54.7 | 24,684 |
