# Supplementary material for: Opioid overdose and naloxone administration knowledge and perceived competency in a probability sample of Indiana urban communities with large Black populations
Source: PLoS One. 2025 Jul 15;20(7):e0328444. doi: 10.1371/journal.pone.0328444 (PMC12262839; doi:10.1371/journal.pone.0328444)
Supplement: Attachment — Submitted filename: PONE-D-24-50788_R1_reviewer-tcsw22.pdf [file pone.0328444.s008.pdf]

# PLOS ONE

## Opioid Overdose and Naloxone Administration Knowledge and Perceived Competency in A Probability Sample of Indiana Urban Communities with Large Black Populations

--Manuscript Draft--

| | |
|---|---|
| **Manuscript Number:** | PONE-D-24-50788R1 |
| **Article Type:** | Research Article |
| **Full Title:** | Opioid Overdose and Naloxone Administration Knowledge and Perceived Competency in A Probability Sample of Indiana Urban Communities with Large Black Populations |
| **Short Title:** | Community Overdose Knowledge and Response Competency |
| **Corresponding Author:** | Dong-Chul Seo, Ph.D.<br>Indiana University Bloomington School of Public Health<br>Bloomington, Indiana UNITED STATES OF AMERICA |
| **Keywords:** | overdose; opioid; naloxone; Knowledge; competency; urban community |
| **Abstract:** | Background: Despite widespread naloxone distribution efforts, opioid-involved overdose death rates remain high in the United States, with recent data indicating disproportionately greater increases among Black residents. Understanding knowledge and perceived competency regarding opioid overdose and naloxone administration is essential for efficacious targeted public health interventions.<br>Methods: We conducted a household probability survey from March to May 2023 among 772 urban residents across eight Indiana zip code areas with high (> 40%) proportions of Black residents. Multilevel modeling was used to examine individual- and community-level factors associated with opioid overdose knowledge and perceived competency, using adapted items from the Opioid Overdose Knowledge Scale (OOKS) and Opioid Overdose Attitudes Scale (OOAS).<br>Results: Race, sex, household income, education, time lived in the community, and history of opioid overdose significantly predicted knowledge scores. White participants scored higher (mean 6.65) than Black participants (5.70) ($p < 0.001$). A significant cross-level interaction was found, with Black residents living in high-poverty areas scoring lower than White counterparts ( = 1.06, $p = 0.039$). In contrast, perceived competency was primarily associated with age and personal history of overdose.<br>Conclusions: This study provides novel evidence about how opioid overdose knowledge is associated with multi-level variable interactions, such as between community-level factors (poverty) and individual-level factors (race). Findings highlight the potential value of developing culturally and structurally responsive opioid education interventions, particularly for socioeconomically disadvantaged Black communities. |
| **Order of Authors:** | Shin Hyung Lee |
| | Jon Agley, PhD |
| | Vatsla Sharma |
| | Francesca Williamson, PhD |
| | Pengyue Zhang, PhD |
| | Dong-Chul Seo, Ph.D. |
| **Response to Reviewers:** | Editor:<br>1. Please ensure that your manuscript meets PLOS ONE's style requirements, including those for file naming. The PLOS ONE style templates can be found here:<br>We have edited to follow the style requirements.<br><br>2. Please provide an amended statement that declares *all* the funding or sources of support (whether external or internal to your organization) received during this study, as detailed online in our guide for authors at http://journals.plos.org/plosone/s/submit-now. Please also include the statement “There was no additional external funding received for this study.” in your updated Funding Statement. Please include your amended Funding Statement within your cover letter. We will change the online submission form on your behalf. |

Powered by Editorial Manager® and ProduXion Manager® from Aries Systems Corporation

Done as requested.

3. We note that you have indicated that there are restrictions to data sharing for this study. For studies involving human research participant data or other sensitive data, we encourage authors to share de-identified or anonymized data. However, when data cannot be publicly shared for ethical reasons, we allow authors to make their data sets available upon request. For information on unacceptable data access restrictions, please see http://journals.plos.org/plosone/s/data-availability#loc-unacceptable-data-access-restrictions. Before we proceed with your manuscript, please address the following prompts: If there are ethical or legal restrictions on sharing a de-identified data set, please explain them in detail (e.g., data contain potentially identifying or sensitive patient information, data are owned by a third-party organization, etc.) and who has imposed them (e.g., a Research Ethics Committee or Institutional Review Board, etc.). Please also provide contact information for a data access committee, ethics committee, or other institutional body to which data requests may be sent. Please update your Data Availability statement in the submission form accordingly.
We will make datasets available upon request. We have updated Data Availability Statement as requested.

4. Please remove any funding-related text from the manuscript and let us know how you would like to update your Funding Statement.
In response to Request #2 above, we have already amended our Funding Statement and included it within our revised cover letter. We have removed all funding-related text from the abstract and main narrative.

5. Please include captions for your Supporting Information files at the end of your manuscript, and update any in-text citations to match accordingly. Please see our Supporting Information guidelines for more information:
http://journals.plos.org/plosone/s/supporting-information.
Done as requested.

Reviewer #1:
The response rate is relatively low 23%
While the overall response rate of 23% is relatively low, it is consistent with the rates typically reported in contemporary household-based probability surveys using mail and web modes without in-person follow-up. Recent reports from the American Association for Public Opinion Research (AAPOR) attribute declining response rates to broader trends such as survey fatigue and reduced willingness to participate. To minimize potential nonresponse bias, we employed probability sampling, survey weighting, and multiple follow-up strategies, including reminder letters, mailed paper surveys, and incentives. Furthermore, the demographic composition of our final analytic sample closely matched census benchmarks for the target communities, suggesting limited risk of nonresponse bias.
This is also supported by the findings from a replicated methodological study by Keeter and his colleagues (Keeter et al., 2006) which compared standard recruitment (25% response rate) with a rigorous recruitment (50% response rate) using random-digit-dial (RDD) methods. Across 77 of 84 variables, the surveys produced statistically indistinguishable results, indicating that unit nonresponse within this response rate range did not meaningfully compromise data quality (Keeter et al., 2006). The study also found that even respondents hardest to reach or those who partially completed interviews did not differ systematically in key behaviors or attitudes (Keeter et al., 2006) - further supporting the robustness of survey estimates despite modest response rates.
[Keeter, S., Kennedy, C., Dimock, M., Best, J., & Craighill, P. (2006). Gauging the impact of growing nonresponse on estimates from a national RDD telephone survey. International Journal of Public Opinion Quarterly, 70(5), 759-779]

What are the steps taken to calculate demographic matching?
We used four zip-code level demographic matching variables: Black racial proportion, families living in poverty, population size, and proportion of children eligible for free/reduced lunch. Detailed information has been provided in text (Lines 144 – 152).

You used OOKS and OOAS without any clarifications for the psychometric validation?
We apologize for the confusion. While OOKS (on knowledge) and OOAS (on attitudes)

Powered by Editorial Manager® and ProduXion Manager® from Aries Systems Corporation

are companion scales, we only used OOKS to measure knowledge. As requested, we have computed and reported the psychometric properties of both the knowledge scale and the perceived level of competency in managing opioid overdoses scale in our sample. The Cronbach's alpha for the perceived level of competency was 0.87 but it was 0.41 for the knowledge scale. Although the Cronbach's alpha in our sample was modest for the knowledge scale, we have provided justification for its acceptability by citing two supporting articles. Specifically, one should not expect a knowledge test to function in a similar psychometric manner to a scale intended to measure a singular construct, since not all knowledge items measure the same constructs. We have revised the text accordingly (Lines 165 – 170).

There is a significant finding that Black participants in high-poverty areas had lower knowledge scores than their White counterparts. However, the mechanisms or contributing factors are not well explored.
We appreciate the comments. We agree that the mechanisms underlying the observed differences are important to explore. However, as this study represents baseline, exploratory findings within a larger intervention project, no prior analyses among Indiana urban residents were available to further investigate potential contributing factors. We have clarified this in the revised manuscript and highlighted that while the results suggest important differences, identifying specific mechanisms will require further longitudinal and qualitative research (Lines 365 – 368).

In limitation section you should mention social bias and lack of qualitative data and using only 10 knowledge and 7 competency items
As requested, we have added the limitation of potential social desirability bias and the limited number of knowledge and competency items, and the absence of qualitative data (Lines 361 – 368).

The method section should include response rate the different approach
As requested, we have revised the Methods section to explicitly report the overall response rate (23%) and added a detailed description of the sequential survey administration approach, including mailings, reminders, and incentive use (Lines 153 – 162). To further clarify the data collection process, we have also created and included a flowchart (Figure S2) that visually summarizes the survey administration timeline and participation outcomes.

Table 2 is dense
As requested, we have revised Table 2 to streamline its content and improve readability. Additionally, we have moved specific survey items to Table S4 to make the main table more concise while still providing detailed information for readers who wish to review it.

Add some diagrams or graphs
Done as suggested. As mentioned above, we have developed a flowchart illustrating the sample selection and survey administration process to enhance clarity. The flowchart has been added as Figure S2 in the revised manuscript.

Language and grammar editing
Done as suggested.

Reviewer #2:
In abstract should be explained briefly related to IMRAD, furthermore in abstract also explained what becomes Novelty.
As requested, we have revised the Abstract to follow the IMRAD structure more clearly, providing distinct sections for the background, methods, results, and conclusions. In addition, we have added a sentence highlighting the novelty of the study, specifically noting how our findings contribute to new evidence regarding the interaction between structural factors (e.g., community poverty) and race in shaping opioid overdose knowledge (Lines 42 – 62).

In the introduction, I have not found any Research Gap, so researchers need to improve; where from the Research Gap, researchers can give something new from this research, and I have not found what is offered by researchers as Novelty. Literature

Powered by Editorial Manager® and ProduXion Manager® from Aries Systems Corporation

review explains how previous studies work, and you can tell what you find in previous research and what you find new from this research.
As requested, we have revised the Introduction and literature review section to clarify the research gap and the novelty of the study (Lines 84 – 86 & 111 – 118).

In the methodology may need to be explained in more detail related to the research subject so that it becomes clear who is the subject.
Done as suggested (Lines 144 – 152)

In results and discussion should give an idea of the basis of the research's purpose and discuss in-depth the findings in the field, and from there, researchers can give something new from this research.
As requested, we have revised the opening section of the Discussion section to better connect our findings back to the study's research purpose, emphasize the novelty of our work, and more clearly explain how our findings contribute new insights to the field (Lines 286 – 298).

Please add Conclusions must certainly be able to answer from the purpose of this study.
Done as suggested (Lines 369 – 380)

Reviewer #3:
Thank you for providing me the opportunity to review this manuscript. Overall I found this manuscript to be an interesting read, describing the disparity in knowledge of naloxone and opioid overdoses and be impacted by systemic issues related to race. It is a timely reminder that we all need to do more in our work to ensure the right messages are reaching the right people, regardless of race or other factors.
Thank you for your thoughtful and encouraging feedback.

I think there are two areas of improvement needed for this manuscript: 1) being concise, and 2) clearly describing all statistical analyses completed.
In view of the reviewer comments, we have revised the manuscript to be more concise and have clarified the description of all statistical analyses.

I am a firm believer of using less words where possible, and I would suggest the authors look at areas where they could reduce the word count. At times, some sections felt overly described. Perhaps the section that stood out to me the most was when describing listwise deletion and why it was used. I would assume most people reading would understand listwise deletion, and that it's use when describing could be said it in fewer words. As another suggestion to shorten the methods, is provide the survey instrument as a supplementary to reduce the word count in the methods when describing the survey. I appreciate the authors have submitted to a non-addiction or drug and alcohol journal, and therefore at times need to describe certain concepts in greater details.
As requested, we have revised the manuscript to make it concise where possible, including the explanation of listwise deletion. Additionally, we have moved the detailed description of the survey procedure to Figure S2, as suggested.

I was confused as the methods made no mention of using chi-square or t-test analyses, and yet table 1 and table 2 have results from these tests. It was not until i noticed the footnotes of each table that i realized these tests were completed for each table. It would be important to ensure this is adequately described within the statistical analyses section. Did the authors utilize a Bonferroni correction for the chi-square test? Are there further analyses completed that as a reader I am not aware of?
We apologize for the lack of clarity in the Methods section. We have revised the Statistical Analyses subsection to explicitly state that we conducted adjusted chi-square tests with design-based Rao-Scott corrections for categorical variables and design-based F-tests for continuous variables in Tables 1 and 2 (Lines 214 – 217). No Bonferroni correction was applied for these descriptive comparisons for two reasons. First, even with Bonferroni adjustment with 10 pairwise comparisons (using 0.005 rather than 0.05 as the p value threshold), all the reported statistical significance stays unchanged. Second, these analyses were intended to characterize the (sub)samples rather than hypotheses-driven statistical tests.

Powered by Editorial Manager® and ProduXion Manager® from Aries Systems Corporation

| | |
|---|---|
| | Reviewer #4:<br>The abstract is intelligible and accurately describes the objective and results obtained. It is a sufficient summary of the contents of the paper. In the abstract, the results should be explored. The CONCLUSIONS could be directly related to the results found in the study.<br>Thank you for the thoughtful feedback. As suggested, we have revised the abstract to explain the results and to ensure the conclusions are directly tied to the findings of the study.<br><br>METHODS: The methods were described and organized in sufficient detail. However, the chapter is quite long. I would suggest to the authors to summarize it. A suggestion would be to present another flowchart with the methodological organization.<br>As suggested, we have summarized the methods section to improve clarity and conciseness and have included an additional flowchart in Figure S2.<br><br>RESULTS: The results are interesting. The authors clearly highlighted the information collected using the methods described to meet the study objectives. The tables with the presentation of the results are organized and clear. The authors identify the limitations of the study in the end of the manuscript, but it is necessary to indicate suggestions for future research.<br>As suggested, we have provided suggestions for future research (Lines 378 – 380).<br><br>CONCLUSION. The authors could present a clear and objective conclusion.<br>As suggested, we have revised the Conclusion section to present a clearer and more objective summary of the findings (Lines 369 – 380).<br><br>REFERENCES: The authors cite appropriate and actual papers for comments/discussion made<br>Thank you. |
| **Additional Information:** | |
| **Question** | **Response** |
| **Financial Disclosure**<br><br>Enter a financial disclosure statement that describes the sources of funding for the work included in this submission. Review the submission guidelines for detailed requirements. View published research articles from *PLOS ONE* for specific examples.<br><br>This statement is required for submission and **will appear in the published article** if the submission is accepted. Please make sure it is accurate. | Yes |

| | |
|---|---|
| **Funded studies**<br>Enter a statement with the following details:<br>• Initials of the authors who received each award<br>• Grant numbers awarded to each author<br>• The full name of each funder<br>• URL of each funder website<br>• Did the sponsors or funders play any role in the study design, data collection and analysis, decision to publish, or preparation of the manuscript?<br><br>Did you receive funding for this work? | |
| Please add funding details.<br>as follow-up to "**Financial Disclosure**<br><br>Enter a financial disclosure statement that describes the sources of funding for the work included in this submission. Review the submission guidelines for detailed requirements. View published research articles from *PLOS ONE* for specific examples.<br><br>This statement is required for submission and **will appear in the published article** if the submission is accepted. Please make sure it is accurate.<br><br>**Funded studies**<br>Enter a statement with the following details:<br>• Initials of the authors who received each award<br>• Grant numbers awarded to each author<br>• The full name of each funder<br>• URL of each funder website<br>• Did the sponsors or funders play any role in the study design, data collection and analysis, decision to publish, or preparation of the manuscript?<br><br>Did you receive funding for this work?" | This Project was supported by the Office of Minority Health (OMH) of the U.S. Department of Health and Human Services (HHS) as part of a financial assistance award (CPIMP221346). The contents are those of the authors and do not necessarily represent the official views of, nor an endorsement, by OMH/OASH/HHS, or the U.S. Government. For more information, please visit https://www.minorityhealth.hhs.gov/. There was no additional external funding received for this study. |
| Please select the country of your main research funder (please select carefully as in some cases this is used in fee calculation). | UNITED STATES - US |

| | |
|---|---|
| as follow-up to "**Financial Disclosure**<br><br>Enter a financial disclosure statement that describes the sources of funding for the work included in this submission. Review the submission guidelines for detailed requirements. View published research articles from *PLOS ONE* for specific examples.<br><br>This statement is required for submission and **will appear in the published article** if the submission is accepted. Please make sure it is accurate.<br><br>**Funded studies**<br>Enter a statement with the following details:<br>• Initials of the authors who received each award<br>• Grant numbers awarded to each author<br>• The full name of each funder<br>• URL of each funder website<br>• Did the sponsors or funders play any role in the study design, data collection and analysis, decision to publish, or preparation of the manuscript?<br><br>Did you receive funding for this work?" | |
| **Competing Interests**<br><br>Use the instructions below to enter a competing interest statement for this submission. On behalf of all authors, disclose any competing interests that could be perceived to bias this work—acknowledging all financial support and any other relevant financial or non-financial competing interests.<br><br>This statement is required for submission and **will appear in the published article** if the submission is accepted. Please make sure it is accurate and that any funding sources listed in your Funding Information later in the submission form are also declared in your Financial Disclosure statement. | The authors have declared that no competing interests exist. |

*Powered by Editorial Manager® and ProduXion Manager® from Aries Systems Corporation*

| | |
|---|---|
| View published research articles from *PLOS ONE* for specific examples.<br><br>**NO authors have competing interests**<br><br>Enter: *The authors have declared that no competing interests exist.*<br><br>**Authors with competing interests**<br><br>Enter competing interest details beginning with this statement:<br><br>*I have read the journal's policy and the authors of this manuscript have the following competing interests: [insert competing interests here]*<br><br>* typeset | |
| **Ethics Statement**<br><br>Enter an ethics statement for this submission. This statement is required if the study involved:<br><br>• Human participants<br>• Human specimens or tissue<br>• Vertebrate animals or cephalopods<br>• Vertebrate embryos or tissues<br>• Field research<br><br>Write "N/A" if the submission does not require an ethics statement.<br><br>General guidance is provided below. Consult the submission guidelines for detailed instructions. **Make sure that all information entered here is included in the Methods section of the manuscript.** | This study was approved by the Indiana University Institutional Review Board (IUIRB), which determined that the research met the requirements for federal and University criteria for exemption (approved protocol ID: 16953). Informed consent was obtained for all participants. Waiver of documentation of consent was approved by the IUIRB. |

Powered by Editorial Manager® and ProduXion Manager® from Aries Systems Corporation

| | |
|---|---|
| **Format for specific study types**<br><br>**Human Subject Research (involving human participants and/or tissue)**<br>• Give the name of the institutional review board or ethics committee that approved the study<br>• Include the approval number and/or a statement indicating approval of this research<br>• Indicate the form of consent obtained (written/oral) or the reason that consent was not obtained (e.g. the data were analyzed anonymously)<br><br>**Animal Research (involving vertebrate animals, embryos or tissues)**<br>• Provide the name of the Institutional Animal Care and Use Committee (IACUC) or other relevant ethics board that reviewed the study protocol, and indicate whether they approved this research or granted a formal waiver of ethical approval<br>• Include an approval number if one was obtained<br>• If the study involved *non-human primates*, add *additional details* about animal welfare and steps taken to ameliorate suffering<br>• If anesthesia, euthanasia, or any kind of animal sacrifice is part of the study, include briefly which substances and/or methods were applied<br><br>**Field Research**<br><br>Include the following details if this study involves the collection of plant, animal, or other materials from a natural setting:<br>• Field permit number<br>• Name of the institution or relevant body that granted permission | |
| **Data Availability**<br><br>Authors are required to make all data underlying the findings described fully available, without restriction, and from the time of publication. PLOS allows rare exceptions to address legal and ethical concerns. See the PLOS Data Policy and FAQ for detailed information. | No - some restrictions will apply |

| | |
|---|---|
| A Data Availability Statement describing where the data can be found is required at submission. Your answers to this question constitute the Data Availability Statement and **will be published in the article**, if accepted.<br><br>**Important:** Stating 'data available on request from the author' is not sufficient. If your data are only available upon request, select 'No' for the first question and explain your exceptional situation in the text box.<br><br>Do the authors confirm that all data underlying the findings described in their manuscript are fully available without restriction? | |
| **Describe where the data may be found in full sentences. If you are copying our sample text, replace any instances of XXX with the appropriate details.**<br><br>• If the data are **held or will be held in a public repository**, include URLs, accession numbers or DOIs. If this information will only be available after acceptance, indicate this by ticking the box below. For example: *All XXX files are available from the XXX database (accession number(s) XXX, XXX.*).<br>• If the data are all contained **within the manuscript and/or Supporting Information files**, enter the following: *All relevant data are within the manuscript and its Supporting Information files.*<br>• If neither of these applies but you are able to provide **details of access elsewhere**, with or without limitations, please do so. For example:<br><br>*Data cannot be shared publicly because of [XXX]. Data are available from the XXX Institutional Data Access / Ethics Committee (contact via XXX) for researchers who meet the criteria for access to confidential data.*<br><br>*The data underlying the results presented in the study are available from (include the name of the third party* | Datasets can be available upon request. The request can be submitted to the Human Research Protection Program (HRPP) Office for Research Compliance, Indiana University, 509 E. Third St., Bloomington, Indiana 47401, U.S.A. Once the HRPP receives such a request, the committee will work with the investigators to deidentify data by removing zip code and race/ethnicity information before any release and obtain approval from the Indiana University Institutional Review Board on the appropriateness of the deidentification. The reason for not sharing the data publicly is mainly because the data include personally identifiable information and illicit drug use information while data were collected from only small specific geographical areas (8 zip code areas). |

Powered by Editorial Manager® and ProduXion Manager® from Aries Systems Corporation

| | |
|---|---|
| *and contact information or URL).*<br>• This text is appropriate if the data are owned by a third party and authors do not have permission to share the data.<br><br>* typeset | |
| Additional data availability information: | |

Powered by Editorial Manager® and ProduXion Manager® from Aries Systems Corporation

Revised Manuscript - clean copy

Click here to access/download;Manuscript;Revised Manuscript Clean_PLoS One_0506.docx

# Opioid Overdose and Naloxone Administration Knowledge and Perceived Competency in A Probability Sample of Indiana Urban Communities with Large Black Populations

**List of authors:**

Shin Hyung Lee[1]; Jon Agley[1] ; Vatsla Sharma[2]; Francesca Williamson[3]; Pengyue Zhang[4]; Dong-Chul Seo[1*]

**Affiliations and addresses:**

[1] Department of Applied Health Science, Indiana University School of Public Health-Bloomington, IN, U.S.A.

[2] O'Neill School of Public and Environmental Affairs, Indiana University-Bloomington, IN, U.S.A.

[3] Department of Learning Health Sciences, University of Michigan Medical School, Ann Arbor, MI, U.S.A.

[4] Department of Biostatistics and Health Data Science, Indiana University School of Medicine, Indianapolis, IN, U.S.A.

***Corresponding author:**

Dong-Chul Seo, PhD, FNYAM, FAAHB
Professor
Suite 116, 1025 E. 7th Street
Department of Applied Health Science
Indiana University School of Public Health-Bloomington
Bloomington, IN 47405-7109, U.S.A.
E-mail: seo@iu.edu, Phone: 1(812) 855-9379, Fax: 1(812) 855-3936

**Running head:**

Community Overdose Knowledge and Response Competency

**Word count:**

Abstract – 223 words
Main text – 3872words (excluding references and tables)
Number of tables – 4
Number of figures – 0
Number of references – 54

**Conflict of interest**

All authors declare that they have no conflicts of interest.

# Abstract

**Background:** Despite widespread naloxone distribution efforts, opioid-involved overdose death rates remain high in the United States, with recent data indicating disproportionately greater increases among Black residents. Understanding knowledge and perceived competency regarding opioid overdose and naloxone administration is essential for efficacious targeted public health interventions.

**Methods:** We conducted a household probability survey from March to May 2023 among 772 urban residents across eight Indiana zip code areas with high (> 40%) proportions of Black residents. Multilevel modeling was used to examine individual- and community-level factors associated with opioid overdose knowledge and perceived competency, using adapted items from the Opioid Overdose Knowledge Scale (OOKS) and Opioid Overdose Attitudes Scale (OOAS).

**Results:** Race, sex, household income, education, time lived in the community, and history of opioid overdose significantly predicted knowledge scores. White participants scored higher (mean 6.65) than Black participants (5.70) ($p < 0.001$). A significant cross-level interaction was found, with Black residents living in high-poverty areas scoring lower than White counterparts ($\beta = 1.06$, $p = 0.039$). In contrast, perceived competency was primarily associated with age and personal history of overdose.

**Conclusions:** This study provides novel evidence about how opioid overdose knowledge is associated with multi-level variable interactions, such as between community-level factors (poverty) and individual-level factors (race). Findings highlight the potential value of developing culturally and structurally responsive opioid education interventions, particularly for socioeconomically disadvantaged Black communities.

**Keywords**: overdose; opioid; naloxone; knowledge; competency; urban community.

# Introduction

With the recent proliferation of illicit synthetic opioids, including fentanyl and its analogues, Indiana, like other states, is facing a significant public health challenge from opioid-involved overdoses (1, 2). In 2022, Indiana ranked as the 13th highest state in drug overdose mortality nationwide, with a rate of 41.0 deaths per 100,000 population. Moreover, Indiana recorded 2,072 deaths attributed to opioid overdoses, comprising 77% of the overall drug overdose fatalities within the state in 2022 (3). In Indianapolis, the most populous city in Indiana, there were 195 fentanyl-related drug overdose deaths in 2018, and this number had increased to 799 deaths in 2021 (1).

Researchers have expressed concern that the increase in opioid-involved overdoses and fatalities is disproportionately affecting marginalized communities, with Black Americans in urban settings particularly impacted (4-6). In 2018 and 2019, the age-adjusted opioid overdose death rate per 100,000 people was higher for White people (18.8 and 19.2, respectively) than for Black people (14.1 and 17.3, respectively), but in 2020, data began showing a higher death rate for Black people than White people and diverging trend lines. By 2022, the opioid (7) overdose death rate for Black people was 36.6 and the rate for White people was 27.6 (3). Gondré-Lewis et al. (2023) also reported that several major urban areas, including Baltimore, Chicago, Detroit, and Philadelphia, with large Black populations, had markedly higher opioid-related death rates compared to rural areas (5). Similarly, Black communities in Indiana experienced the largest increase in opioid-related overdose deaths in recent years (8). However, the mechanisms behind these racial differences for prevalence rates and trends, especially in urban Indiana settings, remain poorly understood.

Administration of naloxone to reverse opioid overdoses is one key area where data suggest population-level differences that may be associated with opioid-related death rates. Naloxone, an opioid antagonist, is a safe and effective medication that can be easily administered to anyone undergoing an opioid overdose to reverse that overdose (9, 10). Research suggests that the incidence of fatal opioid overdoses could decrease if community members have access to naloxone and are educated about opioid-involved overdose and naloxone administration (11-13). Yet, in their Baltimore city based study, Dayton et al. (2020) found that White participants had significantly higher odds of receiving naloxone training and using naloxone compared to Black participants (14). In their study on drug overdose deaths, Takemoto et al. (2024) and Ray et al. (2019) found racial differences in naloxone administration, with White decedents more likely to have had naloxone administered compared to Black decedents (15, 16). At the same time, most U.S. states have enacted legislation to expand its accessibility and utilization among laypersons to address public health emergency of opioid-involved fatalities (9, 12, 17). In addition, the U.S. Food and Drug Administration recently approved some formulations of naloxone for over-the-counter sale nationwide (18).

The State of Indiana has undertaken substantial efforts to expand access to naloxone in recent years, and more than 578,000 doses of naloxone have been distributed throughout Indiana as part of these efforts since 2020 (19). Still, there are multiple factors that may hinder timely naloxone administration at overdose scenes, such as lack of knowledge about opioid overdose and naloxone administration methods (20). Such barriers to timely naloxone administration to reverse opioid-involved overdoses are complex (20) and may be related to insufficient understanding of opioid overdose and naloxone administration (21, 22). Courtney et al. (2023)

found that lack of knowledge about opioid overdose and naloxone administration among laypersons were among the major barriers for using naloxone at overdose scenes (21).

Despite the importance of reducing rates of opioid-involved overdoses, no prior studies have specifically assessed opioid overdose knowledge and perceived naloxone competency among Black urban residents in Indiana. In order to develop effective interventions, it is necessary to better understand the individual- and community-level factors associated with opioid overdose knowledge and perceived naloxone competency. By leveraging recent probability samples across multiple Indiana cities, we aim to provide baseline data critical for informing community-based interventions designed to better ensure that all communities have access to effective means of reducing opioid-involved overdose fatality rates.

Accordingly, the objectives of this study were (1) to measure the level of knowledge and perceived competency regarding opioid overdose and naloxone administration in recent probability samples of Black urban communities in Indiana, (2) investigate racial and other sociodemographic correlates of knowledge and perceived competency, and (3) assess possible relationships, if any, between community-level factors (e.g., proportion of Black residents in the community, community-level income and employment rates) and knowledge and perceived competency.

# Methods

## Ethics statement

This study was approved by the Indiana University Institutional Review Board (IUIRB), which determined that the research met the federal and university criteria for exemption (approved protocol ID: 16953). Informed consent was obtained for all participants. Waiver of documentation of consent was approved by the IUIRB.

## Sample and administration

This study was part of the Multi-Sector and Multi-Level Community-Driven Approaches to Remove Structural Racism and Overdose Deaths in Black Indianapolis Communities (MACRO-B), a project that includes opioid overdose and naloxone administration community education programming to reduce opioid overdose deaths in Black Indianapolis communities. As part of the project, data were collected from March 2023 to May 2023 via mail and web-based household probability community surveys with samples of non-institutionalized adults aged 18 years or older. Demographic characteristics of survey respondents are shown in Table S1. Data collection was managed by the Indiana University Center for Survey Research. Probability sampling was used to select respondents representative of eight urban zip code areas in Indiana with Black populations greater than 40%, including inner-city Indianapolis (46202, 46205, 46208, and 46218) and northern Indiana communities—Gary (46408), Merrillville (46410), South Bend (46628), and Fort Wayne (46806). The target intervention communities in Indianapolis were selected based on the high percentage of Black residents and high rates of opioid overdose events and deaths. The comparison communities in northern Indiana were selected by zip-code-level matching on the proportion of Black population, percentages of families living in poverty, population size, and the proportion of children eligible for free/reduced lunch, using the data from the United States Census Bureau and Indiana State Department of Health overdose surveillance reports (7, 19). The intervention and comparison communities were similar in terms of the four matching variables while they were geographically distant (i.e., 125 to 150 miles away).

The study adhered to the guidelines established by the American Association for Public Opinion Research (AAPOR) (23). Survey administration proceeded in sequential stages. On day

1, households were mailed a preliminary letter containing a $1 pre-incentive and instructions for completing an online survey, along with notification that a $20 gift card would be provided upon completion. Reminder letters were sent to non-respondents on day 10 and day 20, with the second reminder including a paper version of the survey and a self-addressed, stamped return envelope. A third reminder with a paper version of the survey was mailed on day 35, followed by a final reminder on days 50-55, encouraging participation either online or by mail. The overall response rate was 23%, calculated using AAPOR Response Rate Formula #4 (23). A detailed summary of the survey administration timeline is available in Figure S2.

# Measures

## Knowledge on opioid overdose and naloxone administration

To assess knowledge, 10 items assessing opioid overdose knowledge were adapted from the Opioid Overdose Knowledge Scale (OOKS) (24). In the current sample, the internal consistency reliability of the 10-item knowledge scale was modest (Cronbach's alpha = 0.41). This is consistent with prior research suggesting that lower Cronbach's alpha values are acceptable for knowledge-based assessments, particularly when measuring diverse domains of factual information (25, 26). The questionnaire included 6 true/false items and 4 multiple-choice items designed to test basic knowledge about identifying the risks, signs, and symptoms of opioid overdose, as well as understanding the proper use of naloxone (see Table S3 for these items). Each correct answer earned one point, resulting in a possible score range of 0 to 10 for knowledge on opioid overdose and naloxone administration. We calculated mean scores by race, ensuring that all scores were weighted accordingly.

## Perceived competency to manage opioid overdoses

To assess the perceived level of competency in managing opioid overdoses, we asked the survey respondents to rate their agreement with 7 items on a Likert-type scale ranging from 1 to 5 (see Table S3 for these items). These items were also derived from the Opioid Overdose Knowledge Scale (OOKS) and the Opioid Overdose Attitudes Scale (OOAS) (24). Each item was scored individually, and appropriate reverse coding was made so that a higher score always indicated a higher perceived level of competency (Cronbach's alpha = 0.87). We then calculated the weighted mean scores of all 7 items by race.

## Individual-level and zip code-level predictors

One of the key variables in this study was race. In analyses, variable responses were dummy coded as White, Black, and Other. Due to the small sample sizes for specific groups (Asian = 22, American Indian or Alaska Native = 21, and Native Hawaiian or Pacific Islander = 6), we combined those individuals with the "Other" category to ensure sufficient numbers for meaningful analyses (27). Geographic area was determined by the eight zip code areas, which were then dummy coded by region as inner-city Indianapolis (46202, 46205, 46208, and 46218) and other urban areas such as Gary (46408), Merrillville (46410), South Bend (46628), and Fort Wayne (46806). Several demographic factors were included as covariates: age group (18-24, 25-34, 35-44, 45-54, 55-64, 65-74, and 75+), biological sex at birth (women, men), ethnicity (Non-Latino, Latino), educational level (some college or higher, high school/GED or less), and household income, which had the following categories: less than $15,000; $15,000–$24,999; $25,000–$34,999; $35,000–$49,999; $50,000–$74,999; $75,000–$99,999; and $100,000 or more. For analysis purposes, income was collapsed into three broader categories (over $100,000, $35,000-$99,999, less than $35,000). Other covariates included duration of residence in the community (more than 3 years, 1 to 3 years, less than a year) and history with opioid overdose

(yes/no). The variable for opioid overdose history was defined by whether individual had personally experienced an opioid overdose or had a family member or close friend who died from an opioid overdose. Additionally, zip code-level factors were included such as proportion of Black residents, number of individuals with income below the 200 percent of federal poverty level (FPL<200%), median household income, proportion of individuals with high school or higher educational attainment among those who were 25 years or older, and employment rate. These zip-code level data are shown in Table S4. These factors were obtained from the 2022 American Community Survey 5-year estimates (7).

## Statistical analysis

Out of the 772 survey respondents, 47 cases with missing information were excluded using listwise deletion, resulting in a final analytic sample of 725 respondents. Given the low rate of missingness 6% (28), listwise deletion was appropriate to maintain a consistent analytic sample across variables (29). Descriptive statistics included unweighted frequencies and weighted proportions for demographic characteristics and correct responses on opioid overdose and naloxone administration questions. Weighted mean scores were calculated for perceived competency items. Categorical variables were compared across racial groups using adjusted chi-square tests with design-based Rao-Scott corrections. Continuous variables were compared using design-based F-tests, accounting for the complex survey design.

Multilevel analysis models were then constructed to identify factors associated with two outcomes: (a) knowledge and (b) perceived competency regarding opioid overdose and naloxone administration. Following Lee et al. (2015), nested models were sequentially built, beginning with a null model, then adding individual-level socioeconomic variables, overdose experience,

zip code-level predictors, and cross-level interactions (30). All analyses were conducted using R version 4.3.2 (31).

# Results

## Demographic characteristics

Table 1 provides a breakdown of the demographic and socioeconomic characteristics of the survey respondents categorized by race, including White (n = 359), Black (n = 347), and Other racial groups (n = 60). There were significant differences by race observed for the following variables: ethnicity ($p = 0.002$), age group ($p < 0.001$), educational attainment ($p = 0.001$), household income ($p < 0.001$), and opioid overdose history ($p = 0.003$). Region, sex, and length of time lived in the communities were not significantly different by race.

<< Insert Table 1 about here >>

## Weighted scores for knowledge on opioid overdose and naloxone administration and perceived competency level to manage opioid overdoses by race

Table 2 presents the unweighted frequencies and weighted proportions of responses to the 10 questions on opioid overdose and naloxone administration knowledge, along with the weighted mean scores of 7 items that assess perceived competency in managing opioid overdose. The average score on the 10 questions assessing knowledge on opioid overdose and naloxone administration showed significant differences by race ($p < .001$). White participants had the highest scores (Mean = 6.65, *SD* = 1.86), followed by other racial groups (Mean = 5.75, *SD* = 1.89), and then Black participants (Mean = 5.70, *SD* = 1.79). In contrast, perceived competency to manage opioid overdose, assessed through 7 survey items, did not show significant variation

across racial groups ($p$ = 0.478). White participants had a mean score of 2.95 ($SD$ = 1.05), Black participants scored 2.82 ($SD$ = 0.92), and Other racial groups scored 2.96 ($SD$ = 1.07).

Some individual knowledge questions (components of the overall knowledge score) showed significant variation by racial group, whereas none of the individual weighted mean scores for perceived competence items (components of the overall weighted mean competency score) indicated significant differences among racial groups.

<< Insert Table 2 about here >>

## Factors associated with knowledge on opioid overdose and naloxone administration scores

Table 3 presents the multilevel analysis of factors influencing knowledge about opioid overdose and naloxone administration. The model included both individual and zip code-level predictors, as well as interaction terms. In the model, Black (β = -1.74, $p$ = 0.005) participants had significantly lower opioid overdose and naloxone administration knowledge scores compared to their White counterparts. Education level also played a significant role, with participants having a high school education or less scoring lower than those with some college or higher education (β = -0.31, $p$ = 0.044). Income was another significant predictor; compared to those earning > $100,000, those earning < $100,000 showed lower knowledge scores (β = -0.47, $p$ = 0.009 for those earning $35,000 – $99,999 and β = -0.66, $p$ = 0.002 for those earning less than $35,000). Notably, participants with a history of opioid overdose demonstrated higher knowledge scores (β = 0.83, $p$ = <0.001). For the length of time lived in the community, individuals who lived in their area for one to three years scored lower than those who lived there for more than three years (β = -0.42, $p$ = 0.012). Zip code-level predictors showed no significant relationship with knowledge scores. However, a significant cross-level interaction was found

between Black race and FPL<200%, indicating that Black residents living in poorer neighborhoods displayed lower knowledge scores than White counterparts (β = 1.06, $p = 0.039$). The intraclass correlation coefficient (ICC) for the null model was 0.028, indicating that 2.8% of the total variance in the knowledge level was due to differences between the zip codes.

<< Insert Table 3 about here >>

## Factors associated with perceived competency on opioid overdose and naloxone administration

Table 4 summarizes the multilevel model analysis on perceived competency in managing opioid overdose and administering naloxone. At the individual level, race was not a significant predictor, but age was. The youngest age group (18-24 years) reported significantly lower perceived competency (β = -0.34, $p = 0.013$) compared to the 35-44 age group, as did the oldest groups, aged 65-74 (β = -0.45, $p = 0.001$) and 75+ (β = -0.33, $p = 0.013$). No significant differences were noted for the other age groups. The only other significant predictor of perceived competency was having a personal or familial history of opioid overdose, which was associated with a higher score (β = 0.61, $p < 0.001$). The ICC of the null model was 0.004, indicating that only 0.4% of the variance in perceived competency was due to differences between zip codes. Random effects analysis showed minimal variability across zip codes.

<< Insert Table 4 about here >>

# Discussion

This study is one of the first to examine knowledge and perceived competency regarding opioid overdose and naloxone administration among racially diverse urban residents in Indiana. Although previous research has documented rising overdose deaths among Black Americans

nationally, little was known about how knowledge and readiness to respond to overdoses vary by race and neighborhood conditions at the local level. By using probability samples across major urban areas of Indiana, our study provides novel baseline data that highlights significant differences. White urban residents scored the highest on knowledge items, while Black residents scored the lowest. Furthermore, these differences in level of knowledge were associated with community-level poverty via an interaction effect, with Black residents living in high-poverty neighborhoods showing the lowest scores. These findings reveal important structural and contextual barriers to overdose prevention, and suggest that future overdose prevention efforts that are specifically tailored by race and socioeconomic status may help optimize prevention of fatal opioid-involved overdoses.

Our finding that Black urban residents scored lower on knowledge questions around opioid overdose and naloxone administration compared to White urban residents is consistent with prior research (32, 33). Likewise, the relatively low knowledge scores across all urban Indiana residents in the sample aligns with previous research indicating that despite naloxone's relatively widespread availability, knowledge about naloxone is lacking (22, 34-36). This could likely be improved through peer-led educational initiatives (37-41), which are successful in improving participants' knowledge about opioid overdose and naloxone administration.

Seo et al. (2023) found that Black residents in Indianapolis felt they lacked sufficient educational opportunities regarding opioid overdose and naloxone usage compared to White urban residents (42). However, they expressed a readiness to learn about and carry naloxone for overdose response, particularly when the education and support came from trusted figures within their community such as Black church leaders (42). Addressing the gap observed in this study might be achievable by implementing culturally tailored education programs (43, 44) involving

community leaders who grasp the cultural nuances and can communicate effectively with Black residents about the importance of naloxone administration (45).

Also consistent with previous studies (46, 47), our findings indicated that having a history of opioid overdose, whether personal or through the death of a family member or close friend, was associated with significantly higher knowledge scores. This suggests that firsthand experience or proximity to opioid-related events may serve as a catalyst for increased awareness and understanding (46, 47), potentially due to greater engagement with medical professionals or support services following such events. Interestingly, no significant associations were found between zip code-level predictors and knowledge scores except the poverty level of the community. Research has shown that poverty and socioeconomic status are linked to educational access and  health literacy (48) thereby affecting health outcomes (49). Additionally, aligning with previous studies (47, 50) several socioedemographic factors were significantly related with knowledge scores. Men, individuals with lower household income and educational attainment, and those in lower-income brackets exhibited lower knowledge scores. Strategies such as targeted education, improved access to resources, local partnerships, tailored messaging, financial incentives, and ongoing evaluation, may help address knowledge disparities among men, lower-income, and less-educated groups, though exploring their effectiveness lies beyond the scope of this study. Additionally, we found a cross-level interaction between race and poverty level. This interaction emphasizes the need for interventions that address both race and socioeconomic status such that more Black residents in high-poverty areas are provided with tailored educational opportunities on opioid overdose and naloxone administration.

Regarding perceived competency in managing opioid overdose and naloxone administration, age emerged as a significant predictor, with both the youngest (18-24 years) and

oldest age groups (65-74 and 75 years or older) reporting lower perceived competency. This may indicate a lack of practical experience or exposure to training opportunities among these cohorts. Notably, having an opioid overdose history significantly enhanced perceived competency, aligning with our previous findings regarding knowledge scores. This relationship suggests that experiential learning may play an important role in developing confidence in overdose management. In a study based in Nebraska, Cooper-Ohm et al. (2023) found an insignificant relationship between past history and familiarity with naloxone in cities of Omaha and Lincoln, however a significant relationship was found in regions outside these two cities (51). Furthermore, the ICC results from the null models in this study provide insights into the variability of knowledge and perceived competency related to opioid overdose management and naloxone administration across different urban communities. Specifically, the ICC showed that 2.8% of the variance in knowledge was due to geographical differences. Urban communities with limited access to resources could benefit from educational campaigns and support services that address their specific needs (52). By acknowledging the community context, public health practitioners could develop more effective strategies to share important information about opioid overdose prevention and naloxone use (37, 53, 54). On the other hand, the ICC for perceived competency was only 0.4%, which indicates that differences in perceived competency across zip codes are minimal, regardless of the availability of educational resources in these communities.

The findings of this study should be cautiously interpreted within the context of its limitations. By focusing on specific zip codes and recruiting survey respondents from only those zip codes, there may be limited generalizability, potentially applying only to the settings with similar characteristics and environments. Nonetheless, the chosen zip codes were deliberately selected from urban areas with higher proportions of Black residents and elevated rates of

overdose events and deaths compared to other areas in Indiana, rendering the findings of our study meaningful. Additionally, since the survey relied on self-reported data, it is susceptible to response bias and recall bias. Furthermore, we could not assess temporal relationships due to the cross-sectional nature of the survey. Also, the responses to opioid overdose items may have been subject to social desirability bias, potentially leading to overestimation of perceived competence. While our survey included 10 knowledge items and 7 competency items, these items may not have fully captured the breadth of understanding and skills needed for effective overdose response. Finally, the lack of qualitative data limits our ability to explore the underlying reasons behind observed disparities, such as structural barriers or cultural factors affecting knowledge and competency. Future research should incorporate qualitative approaches to better capture the nuances of opioid overdose knowledge and response competency.

This study aimed to assess the level of knowledge and perceived competency regarding opioid overdose and naloxone administration among urban Indiana residents and to examine racial and community-level differences. Our findings demonstrate that significant gaps in knowledge exist, particularly among Black residents living in high-poverty areas, underscoring the value of tailored education initiatives. While strategies to reduce opioid-involved deaths are multifaceted, ensuring layperson access to naloxone, improving knowledge of overdose recognition and response, and strengthening naloxone administration self-efficacy are critical components. As part of the MACRO-B project, we are currently providing community-based overdose prevention education, naloxone administration training, and naloxone distribution. Moving forward, we plan to conduct longitudinal and qualitative research to better understand the mechanisms underlying these differences and to evaluate the long-term effectiveness and sustainability of our intervention efforts.

## Acknowledgements

This work was supported by the Indiana University Center for Survey Research (CSR). We thank the CSR for assisting in administering community surveys in our target study area which greatly assisted the research although they may not agree with all of the interpretations/conclusions of this paper.

## Funding statement

This project was supported by the Office of Minority Health (OMH) of the U.S. Department of Health and Human Services (HHS) as part of a financial assistance award (CPIMP221346). The contents are those of the authors and do not necessarily represent the official views of, nor an endorsement, by OMH/OASH/HHS, or the U.S. Government. For more information, please visit https://www.minorityhealth.hhs.gov/. There was no additional external funding received for this study.

## Captions for supporting information files

S1 Table. Demographic characteristics of survey respondents: March-May 2023 (N = 772)

S2 Fig. Survey administration timeline and follow-up procedure

S3 Table. Weighted frequencies and percentages for knowledge on opioid overdose and naloxone administration by race: March-May, 2023 (N = 772)

S4 Table. Zip-code level predictors: 2022 American Community Survey 5-year estimates

# References

1. Carter L, McGinty, A., Sloan, L., Malcezewski, M., & Sandusky, G. Manner of Death and Fentanyl Related Drug Overdose Trends in Marion County, Indiana, US from 2018 to 2021. Forensic Medicine and Anatomy Research. 2023;11(2):30-7.
2. (NCHS). NCHS. National Center for Health Statistics (NCHS). Drug Overdose Death in the United States 2002-2022. 2024.
3. (KFF) KFF. Opioid overdose deaths and opioid overdose deaths as a percent of all drug overdose deaths 2023 [Available from: https://www.kff.org/other/state-indicator/opioid-overdose-deaths/.
4. Gibbons JB, Harris SJ, Solomon KT, Sugarman O, Hardy C, Saloner B. Increasing overdose deaths among Black Americans: a review of the literature. Lancet Psychiatry. 2023;10(9):719-26.
5. Gondre-Lewis MC, Abijo T, Gondre-Lewis TA. The Opioid Epidemic: a Crisis Disproportionately Impacting Black Americans and Urban Communities. J Racial Ethn Health Disparities. 2023;10(4):2039-53.
6. Kyei EF, Leveille S. Opioid Misuse and Opioid Overdose Mortality Among the Black Population in the United States: An Integrative Review. Policy Polit Nurs Pract. 2023;24(3):208-18.
7. Bureau UC. American Community Survey (ACS). 2024.
8. (FSSA) IFSSA. Analysis of opioid overdose mortality and vulnerability index in Indiana. 2023.
9. (SAMHSA) SAaMHSA. SAMHSA Opioid Overdose Prevention TOOLKIT. 2018.
10. (NIH). NIoH. Naloxone DrugFacts. National Institute on Drug Abuse (NIDA). 2022.
11. Bennett AS, Bell A, Doe-Simkins M, Elliott L, Pouget E, Davis C. From Peers to Lay Bystanders: Findings from a Decade of Naloxone Distribution in Pittsburgh, PA. J Psychoactive Drugs. 2018;50(3):240-6.
12. Razaghizad A, Windle SB, Filion KB, Gore G, Kudrina I, Paraskevopoulos E, et al. The Effect of Overdose Education and Naloxone Distribution: An Umbrella Review of Systematic Reviews. Am J Public Health. 2021;111(8):1516-7.
13. Lambdin BH, Zibbell J, Wheeler E, Kral AH. Identifying gaps in the implementation of naloxone programs for laypersons in the United States. Int J Drug Policy. 2018;52:52-5.
14. Dayton L, Tobin K, Falade-Nwulia O, Davey-Rothwell M, Al-Tayyib A, Saleem H, et al. Racial Disparities in Overdose Prevention among People Who Inject Drugs. J Urban Health. 2020;97(6):823-30.
15. Takemoto E, Bolton A, Goetz CT. Inequities in naloxone administration among fatal overdose decedents by race and ethnicity in Pennsylvania, 2019-21. Addiction. 2024;119(8):1400-9.
16. Ray B, Lowder E, Bailey K, Huynh P, Benton R, Watson D. Racial differences in overdose events and polydrug detection in Indianapolis, Indiana. Drug Alcohol Depend. 2020;206:107658.
17. Smart R, Pardo B, Davis CS. Systematic review of the emerging literature on the effectiveness of naloxone access laws in the United States. Addiction. 2021;116(1):6-17.
18. (FDA) USFDA. FDA Approves First Over-the-Counter Naloxone Nasal Spray 2023.
19. Recovery N. Progress Report. The Indiana State Government. 2023.

20. Bennett AS, Freeman R, Des Jarlais DC, Aronson ID. Reasons People Who Use Opioids Do Not Accept or Carry No-Cost Naloxone: Qualitative Interview Study. JMIR Form Res. 2020;4(12):e22411.
21. Courtney J, Ferrarotti, A., Tran, T., & Titus-Lay, E. Layperson Knowledge and Perceived Barriers With Obtaining Naloxone. Journal of Contemporary Pharmacy Practice. 2023;70(3):16-26.
22. Schlosser A, Habecker P, Bevins R. Harm reduction in the Heartland: public knowledge and beliefs about naloxone in Nebraska, USA. Harm Reduct J. 2022;19(1):22.
23. (AAPOR). TAAfPOR. Standard Definitions: Final Dispositions of Case Codes and Outcome Rates for Surveys. 10th 2023 [Available from: https://aapor.org/wp-content/uploads/2023/05/Standards-Definitions-10th-edition.pdf.
24. Williams AV, Strang J, Marsden J. Development of Opioid Overdose Knowledge (OOKS) and Attitudes (OOAS) Scales for take-home naloxone training evaluation. Drug Alcohol Depend. 2013;132(1-2):383-6.
25. Streiner DL. tarting at the beginning: An introduction to coefficient alpha and internal consistency. Journal of Personality Assessment. 2003;80(1):99-103.
26. KS T. The Use of Cronbach's Alpha When Developing and Reporting Research Instruments in Science Education. Research in Science Education. 2018;48:1279-96.
27. Flanagin A, Frey T, Christiansen SL, Committee AMAMoS. Updated Guidance on the Reporting of Race and Ethnicity in Medical and Science Journals. JAMA. 2021;326(7):621-7.
28. Dong Y, Peng CY. Principled missing data methods for researchers. Springerplus. 2013;2(1):222.
29. Roderick J. A. Little DBR. Statistical Analysis with Missing Data: John Wiley & Sons, Inc.; 2002.
30. Lee CG, Seo DC, Middlestadt SE, Lin HC. Does the Relationship Between Cigarette Smoking and Other Key Health Behaviors Vary by Geographic Area Among US Young Adults? A Multilevel Analysis. Int J Behav Med. 2015;22(4):481-8.
31. Team RC. R: A Language and Environment for Statistical Computing. R Foundation for Statistical Computing, 2023 [Available from: https://www.R-project.org/.
32. Barboza GE, Angulski K. A descriptive study of racial and ethnic differences of drug overdoses and naloxone administration in Pennsylvania. Int J Drug Policy. 2020;78:102718.
33. Khan MR, Hoff L, Elliott L, Scheidell JD, Pamplin JR, 2nd, Townsend TN, et al. Racial/ethnic disparities in opioid overdose prevention: comparison of the naloxone care cascade in White, Latinx, and Black people who use opioids in New York City. Harm Reduct J. 2023;20(1):24.
34. Bessen S, Metcalf SA, Saunders EC, Moore SK, Meier A, McLeman B, et al. Barriers to naloxone use and acceptance among opioid users, first responders, and emergency department providers in New Hampshire, USA. Int J Drug Policy. 2019;74:144-51.
35. Urmanche AA, Harocopos A. Experiences Administering Naloxone Among People in Different Social Roles: People Who Use Opioids and Family Members and Friends. J Drug Issues. 2023;53(3):475-89.
36. Hohmann LA, Krauss Z, Patel J, Marley GT. Public Perceptions of Community Pharmacy-Based Naloxone Services: A National Cross-Sectional Survey. Pharmacy (Basel). 2022;10(6).

37. Enich M, Flumo R, Campos S, Flores N, Sullivan N, Mellor J, et al. Overdose education and naloxone distribution program design informed by people who use drugs and naloxone distributors. Prev Med Rep. 2023;35:102374.
38. Dos Santos B, Farzan Nipun, R., Maria Subic, A., Kubica, A., Rondinelli, N., Marentette, D., ... & M Orkin, A. Virtual and remote opioid poisoning education and naloxone distribution programs: a scoping review. medRxiv. 2023:11.
39. Owczarzak J, Weicker N, Urquhart G, Morris M, Park JN, Sherman SG. "We know the streets:" race, place, and the politics of harm reduction. Health Place. 2020;64:102376.
40. Hanson BL, Porter RR, Zold AL, Terhorst-Miller H. Preventing opioid overdose with peer-administered naloxone: findings from a rural state. Harm Reduct J. 2020;17(1):4.
41. Neale J, Brown C, Campbell ANC, Jones JD, Metz VE, Strang J, et al. How competent are people who use opioids at responding to overdoses? Qualitative analyses of actions and decisions taken during overdose emergencies. Addiction. 2019;114(4):708-18.
42. Seo DC, Satterfield N, Alba-Lopez L, Lee SH, Crabtree C, Cochran N. "That's why we're speaking up today": exploring barriers to overdose fatality prevention in Indianapolis' Black community with semi-structured interviews. Harm Reduct J. 2023;20(1):159.
43. (SAMHSA) SAaMHSA. Substance Abuse and Mental Health Services Administration: The Opioid Crisis and the Black/African

American Population: An Urgent Issue. . 2020.
44. Hughes M, Suhail-Sindhu S, Namirembe S, Jordan A, Medlock M, Tookes HE, et al. The Crucial Role of Black, Latinx, and Indigenous Leadership in Harm Reduction and Addiction Treatment. Am J Public Health. 2022;112(S2):S136-S9.
45. Winhusen T, Brown, P. L., Ernst, T. I., Ingram, T. I., & Tyehimba, K. A. Increasing naloxone distribution in Black Communities: A case study. . Journal of the National Medical Association. 2024;116(4):424.
46. Buresh M, Gicquelais RE, Astemborski J, Kirk GD, Mehta SH, Genberg BL. Fatal overdose prevention and experience with naloxone: A cross-sectional study from a community-based cohort of people who inject drugs in Baltimore, Maryland. PLoS One. 2020;15(3):e0230127.
47. Resko SM, Pasman E, Hicks DL, Lee G, Ellis JD, O'Shay S, et al. Naloxone Knowledge and Attitudes Towards Overdose Response Among Family Members of People who Misuse Opioids. J Community Health. 2024;49(1):70-7.
48. Raghupathi V, Raghupathi W. The influence of education on health: an empirical assessment of OECD countries for the period 1995-2015. Arch Public Health. 2020;78:20.
49. Schillinger D. Social Determinants, Health Literacy, and Disparities: Intersections and Controversies. Health Lit Res Pract. 2021;5(3):e234-e43.
50. Lazar M, Davenport L. Barriers to Health Care Access for Low Income Families: A Review of Literature. J Community Health Nurs. 2018;35(1):28-37.
51. Cooper-Ohm S, Habecker P, Humeniuk R, Bevins RA. Factors Associated with Gaps in Naloxone Knowledge: Evidence from a 2022 Great Plains Survey. Res Sq. 2023.
52. Rose J, Arikat L, Gusoff G, Pollack CE. Mechanisms to Improve Health Through Community Land Trusts. J Urban Health. 2023;100(2):389-97.
53. Nolen S, Trinidad AJ, Jordan AE, Green TC, Jalali A, Murphy SM, et al. Racial/Ethnic differences in receipt of naloxone distributed by opioid overdose prevention programs in New York City. Res Sq. 2023.

54. Knudsen HK, Walker DM, Mack N, Kinnard EN, Huerta TR, Glasgow L, et al. Reducing perceived barriers to scaling up overdose education and naloxone distribution and medications for opioid use disorder in the United States in the HEALing (Helping End Addiction Long-Term(R)) communities study. Prev Med. 2024;185:108034.

Table 1. Demographic Characteristics by Race: March-May 2023, (N = 772)

| | White (n = 359) | Black (n = 347) | Other (n = 60) | |
|---|---|---|---|---|
| Variable | n (%) | n (%) | n (%) | *p*-value |
| Ethnicity | | | | 0.002 |
| Non-Latine | 323 (87.5) | 330 (95.6) | 43 (76.1) | |
| Latine | 35 (12.5) | 13 (4.4) | 16 (23.9) | |
| Age group | | | | <0.001 |
| 18-24 years | 38 (14.7) | 27 (15.2) | 11 (22.9) | |
| 25-34 years | 82 (30.3) | 36 (9.6) | 10 (16.0) | |
| 35-44 years | 63 (16.6) | 54 (14.4) | 16 (31.8) | |
| 45-54 years | 39 (8.9) | 54 (16.6) | 9 (10.0) | |
| 55-64 years | 47 (12.3) | 67 (22.3) | 6 (6.2) | |
| 65-74 years | 56 (10.6) | 71 (14.7) | 5 (9.6) | |
| 75 years or older | 33 (6.6) | 38 (7.2) | 2 (3.5) | |
| Biological sex at birth | | | | 0.600 |
| Women | 217 (55.0) | 247 (59.2) | 41 (63.7) | |
| Men | 142 (45.0) | 99 (40.8) | 19 (36.3) | |
| Educational attainment | | | | 0.001 |
| Some college or higher | 288 (67.0) | 250 (47.3) | 48 (73.5) | |
| High school /GED or less | 71 (33.0) | 97 (52.7) | 12 (26.5) | |
| Household income | | | | <0.001 |
| Upper, more than $100,000 | 94 (29.8) | 46 (11.5) | 14 (27.3) | |
| Middle, $35,000 - $99,999 | 161 (46.2) | 151 (33.8) | 21 (33.3) | |
| Lower, less than $35,000 | 99 (29.8) | 148 (54.7) | 23 (39.4) | |
| Length of time lived in the communities | | | | 0.554 |
| More than three years | 244 (65.6) | 261 (72.7) | 41 (70.1) | |
| One year to three years | 83 (27.3) | 66 (20.1) | 15 (25.7) | |
| Less than a year | 32 (7.1) | 19 (7.2) | 4 (4.2) | |
| Opioid overdose history[a] | | | | 0.003 |
| No | 264 (69.1) | 275 (78.4) | 53 (93.7) | |
| Yes | 95 (30.9) | 72 (21.6) | 6 (6.3) | |
| Region | | | | 0.996 |
| Indianapolis | 204 (54.3) | 175 (54.8) | 40 (54.8) | |
| Other | 155 (45.7) | 172 (45.2) | 20 (45.2) | |

*Notes.* GED = General Educational Development. Frequencies are unweighted, and proportions are weighted. Frequencies may not sum to the total due to missing observations. Differences between groups were examined via weighted adjusted Chi-Square tests.

[a] Opioid overdose history include individuals with personal opioid overdose experience as well as experiences involving the death of a family member or close friend due to opioid overdose.

Table 2. Weighted Scores for Knowledge on Opioid Overdose and Naloxone Administration and Perceived Competency Level to Manage Opioid Overdoses by Race: March-May, 2023 (N = 772)

| | White (n = 359) | Black (n = 347) | Other (n = 60) | |
|---|---|---|---|---|
| | | Mean (SD) | | *p*-value[a] |
| *Knowledge on Opioid Overdose and Naloxone Administration (10 Questions)* | 6.65 (1.86) | 5.70 (1.79) | 5.75 (1.89) | <0.001 |
| *Perceived Competency to Manage Opioid Overdose (7 items)* | 2.95 (1.05) | 2.82 (0.92) | 2.96 (1.07) | 0.478 |

*Notes.* Frequencies may not sum to the total due to missing observations.
[a] Corresponding p-values were calculated via weighted design-based F-tests.

Table 3. Multilevel Model Analysis of Knowledge on Opioid Overdose and Naloxone Administration (N=723)

| Variable | β | SE | *p*-value |
|---|---|---|---|
| **Fixed Effects** | | | |
| *Individual-level predictors* | | | |
| Intercept | | | |
| Race | | | |
| White | Ref | | |
| Black | **-1.74**** | 0.62 | 0.005 |
| Other | -1.02 | 0.89 | 0.252 |
| Ethnicity | | | |
| Non-Latino | Ref | | |
| Latino | -0.40 | 0.24 | 0.102 |
| Age group | | | |
| 18-24 years | 0.39 | 0.24 | 0.109 |
| 25-34 years | 0.38 | 0.22 | 0.086 |
| 35-44 years | Ref | | |
| 45-54 years | -0.01 | 0.24 | 0.964 |
| 55-64 years | 0.11 | 0.24 | 0.634 |
| 65-74 years | 0.26 | 0.25 | 0.309 |
| 75 years or older | -0.07 | 0.31 | 0.813 |
| Biological sex at birth | | | |
| Women | Ref | | |
| Men | **-0.36**** | 0.13 | 0.007 |
| Educational attainment | | | |
| Some college or higher | Ref | | |
| High school /GED or less | **-0.31*** | 0.16 | 0.044 |
| Household income | | | |
| Upper, more than $100,000 | Ref | | |
| Middle, $35,000 - $99,999 | **-0.47**** | 0.18 | 0.009 |
| Lower, less than $35,000 | **-0.66**** | 0.21 | 0.002 |
| Length of time lived in the communities | | | |
| More than three years | Ref | | |
| One year to three years | **-0.42*** | 0.17 | 0.012 |
| Less than a year | -0.37 | 0.29 | 0.206 |
| Opioid overdose history[a] | | | |
| Yes | **0.83***** | 0.16 | <0.001 |
| No | Ref | | |
| Region | | | |
| Indianapolis | 0.33 | 0.26 | 0.206 |
| Other | Ref | | |
| *Zip code-level predictors* | | | |
| Black proportion | -1.04 | 1.43 | 0.467 |
| FPL < 200%[c] | -0.26 | 0.71 | 0.715 |

| *Cross-level interaction* | | | |
|---|---|---|---|
| Race (Black) * FPL < 200% | **1.06***[*] | 0.51 | 0.039 |

| **Random Effects** | | 95% CI[b] |
|---|---|---|
| $\tau_{00}$ (Level-2) | 0.26 | 0.01 – 0.35 |
| $\sigma^2$ (Level-1) | 1.70 | 1.60 – 1.77 |

*Note.* Ref = reference group. GED = General Educational Development. FPL = Federal Poverty Level. Due to missing data on the variables, the analytical sample was reduced to 723.

[a] Opioid overdose history include individuals with personal opioid overdose experience as well as experiences involving the death of a family member or close friend due to opioid overdose.

[b] 95% profile confidence interval

[c] The number of individuals for FPL<200% was divided by 10,000 to reduce its scale in analysis.

[*] $p < .05$ [**] $p < .01$ [***] $p < .001$.

Table 4. Multilevel Model Analysis of Perceived Competency on Opioid Overdose and Naloxone Administration (N=723)

| Variable | β | SE | *p*-value |
|---|---|---|---|
| **Fixed Effects** | | | |
| *Individual-level predictors* | | | |
| Intercept | | | |
| Race | | | |
| White | Ref | | |
| Black | -0.58 | 0.29 | 0.093 |
| Other[a] | 0.63 | 0.49 | 0.203 |
| Ethnicity | | | |
| Non-Latine | Ref | | |
| Latine | -0.25 | 0.13 | 0.068 |
| Age group | | | |
| 18-24 years | **-0.34**** | 0.13 | 0.013 |
| 25-34 years | -0.11 | 0.12 | 0.392 |
| 35-44 years | Ref | | |
| 45-54 years | -0.15 | 0.14 | 0.264 |
| 55-64 years | -0.04 | 0.13 | 0.764 |
| 65-74 years | **-0.45**** | 0.14 | 0.001 |
| 75 years or older | **-0.33*** | 0.17 | 0.013 |
| Biological sex at birth | | | |
| Women | Ref | | |
| Men | 0.05 | 0.07 | 0.480 |
| Educational attainment | | | |
| Some college or higher | Ref | | |
| High school /GED or less | -0.06 | 0.09 | 0.456 |
| Household income | | | |
| Upper, more than $100,000 | Ref | | |
| Middle, $35,000 - $99,999 | -0.08 | 0.10 | 0.429 |
| Lower, less than $35,000 | -0.08 | 0.11 | 0.469 |
| Length of time lived in the communities | | | |
| More than three years | Ref | | |
| One year to three years | -0.02 | 0.09 | 0.817 |
| Less than a year | -0.09 | 0.16 | 0.571 |
| Opioid overdose history[b] | | | |
| Yes | **0.61***** | 0.09 | <0.001 |
| No | Ref | | |
| Region | | | |
| Indianapolis | -0.07 | 0.10 | 0.491 |
| Other | Ref | | |
| *Zip code-level predictors* | | | |
| Black proportion | 0.24 | 0.55 | 0.664 |
| FPL < 200%[c] | -0.08 | 0.31 | 0.878 |

| | | | |
|---|---|---|---|
| *Cross-level interaction* | | | |
| Race (Black) * FPL < 200% | 0.41 | 0.28 | 0.144 |
| | | | |
| **Random Effects** | | | 95% CI[b] |
| $\tau_{00}$ (Level-2) | | 0.00 | 0.00 – 0.00 |
| $\sigma^2$ (Level-1) | | 0.90 | 0.89 – 0.99 |

*Note.* Ref = reference group. GED = General Educational Development. FPL = Federal Poverty Level. Due to missing data on the variables, the analytical sample was reduced to 723.

[a] Opioid overdose history include individuals with personal opioid overdose experience as well as experiences involving the death of a family member or close friend due to opioid overdose.

[b] 95% profile confidence interval

[c] The number of individuals for FPL<200% was divided by 10,000 to reduce its scale in analysis.

$^{*}$ $p < .05$ $^{**}$ $p < .01$ $^{***}$ $p < .001$

Click here to access/download

**Supporting Information**

supporting_information_PLoS One_0506.docx

Revised Manuscript with Track Changes

# Opioid Overdose and Naloxone Administration Knowledge and Perceived Competency in A Probability Sample of Indiana Urban Communities with Large Black Populations


**List of authors:**

Shin Hyung Lee[1]; Jon Agley[1] ; Vatsla Sharma[2]; Francesca Williamson[3]; Pengyue Zhang[4]; Dong-Chul Seo[1*]

**Affiliations and addresses:**

[1] Department of Applied Health Science, Indiana University School of Public Health-Bloomington, IN, U.S.A.

[2] O'Neill School of Public and Environmental Affairs, Indiana University-Bloomington, IN, U.S.A.

[3] Department of Learning Health Sciences, University of Michigan Medical School, Ann Arbor, MI, U.S.A.

[4] Department of Biostatistics and Health Data Science, Indiana University School of Medicine, Indianapolis, IN, U.S.A.

***Corresponding author:**

Dong-Chul Seo, PhD, FNYAM, FAAHB
Professor
Suite 116, 1025 E. 7th Street
Department of Applied Health Science
Indiana University School of Public Health-Bloomington
Bloomington, IN 47405-7109, U.S.A.
E-mail: seo@iu.edu, Phone: 1(812) 855-9379, Fax: 1(812) 855-3936


**Running head:**

Community Overdose Knowledge and Response Competency

**Word count:**

Abstract – 223 words
Main text – 3872words (excluding references and tables)
Number of tables – 4
Number of figures – 0
Number of references – 54

**Conflict of interest**

All authors declare that they have no conflicts of interest.

# Abstract

**Background:** Despite widespread naloxone distribution efforts, opioid-involved overdose death rates remain high in the United States, with recent data indicating disproportionately greater increases among Black residents. Understanding knowledge and perceived competency regarding opioid overdose and naloxone administration is essential for efficacious targeted public health interventions.

**Methods:** We conducted a household probability survey from March to May 2023 among 772 urban residents across eight Indiana zip code areas with high (> 40%) proportions of Black residents. Multilevel modeling was used to examine individual- and community-level factors associated with opioid overdose knowledge and perceived competency, using adapted items from the Opioid Overdose Knowledge Scale (OOKS) and Opioid Overdose Attitudes Scale (OOAS).

**Results:** Race, sex, household income, education, time lived in the community, and history of opioid overdose significantly predicted knowledge scores. White participants scored higher (mean 6.65) than Black participants (5.70) ($p < 0.001$). A significant cross-level interaction was found, with Black residents living in high-poverty areas scoring lower than White counterparts ($\beta = 1.06$, $p = 0.039$). In contrast, perceived competency was primarily associated with age and personal history of overdose.

**Conclusions:** This study provides novel evidence about how opioid overdose knowledge is associated with multi-level variable interactions, such as between community-level factors (poverty) and individual-level factors (race). Findings highlight the potential value of developing culturally and structurally responsive opioid education interventions, particularly for socioeconomically disadvantaged Black communities.

**Keywords**: overdose; opioid; naloxone; knowledge; competency; urban community.

# Introduction

With the recent proliferation of illicit synthetic opioids, including fentanyl and its analogues, Indiana, like other states, is facing a significant public health challenge from opioid-involved overdoses (1, 2). In 2022, Indiana ranked as the $13^{th}$ highest state in drug overdose mortality nationwide, with a rate of 41.0 deaths per 100,000 population. Moreover, Indiana recorded 2,072 deaths attributed to opioid overdoses, comprising 77% of the overall drug overdose fatalities within the state in 2022 (3). In Indianapolis, the most populous city in Indiana, there were 195 fentanyl-related drug overdose deaths in 2018, and this number had increased to 799 deaths in 2021 (1).

Researchers have expressed concern that the increase in opioid-involved overdoses and fatalities is disproportionately affecting marginalized communities, with Black Americans in urban settings particularly impacted (4-6). In 2018 and 2019, the age-adjusted opioid overdose death rate per 100,000 people was higher for White people (18.8 and 19.2, respectively) than for Black people (14.1 and 17.3, respectively), but in 2020, data began showing a higher death rate for Black people than White people and diverging trend lines. By 2022, the opioid (7) overdose death rate for Black people was 36.6 and the rate for White people was 27.6 (3). Gondré-Lewis et al. (2023) also reported that several major urban areas, including Baltimore, Chicago, Detroit, and Philadelphia, with large Black populations, had markedly higher opioid-related death rates compared to rural areas (5). Similarly, Black communities in Indiana experienced the largest increase in opioid-related overdose deaths in recent years (8). However, the mechanisms behind these racial differences for prevalence rates and trends, especially in urban Indiana settings, remain poorly understood.

Administration of naloxone to reverse opioid overdoses is one key area where data suggest population-level differences that may be associated with opioid-related death rates. Naloxone, an opioid antagonist, is a safe and effective medication that can be easily administered to anyone undergoing an opioid overdose to reverse that overdose (9, 10). Research suggests that the incidence of fatal opioid overdoses could decrease if community members have access to naloxone and are educated about opioid-involved overdose and naloxone administration (11-13). Yet, in their Baltimore city based study, Dayton et al. (2020) found that White participants had significantly higher odds of receiving naloxone training and using naloxone compared to Black participants (14). In their study on drug overdose deaths, Takemoto et al. (2024) and Ray et al. (2019) found racial differences in naloxone administration, with White decedents more likely to have had naloxone administered compared to Black decedents (15, 16). At the same time, most U.S. states have enacted legislation to expand its accessibility and utilization among laypersons to address public health emergency of opioid-involved fatalities (9, 12, 17). In addition, the U.S. Food and Drug Administration recently approved some formulations of naloxone for over-the-counter sale nationwide (18).

The State of Indiana has undertaken substantial efforts to expand access to naloxone in recent years, and more than 578,000 doses of naloxone have been distributed throughout Indiana as part of these efforts since 2020 (19). Still, there are multiple factors that may hinder timely naloxone administration at overdose scenes, such as lack of knowledge about opioid overdose and naloxone administration methods (20). Such barriers to timely naloxone administration to reverse opioid-involved overdoses are complex (20) and may be related to insufficient understanding of opioid overdose and naloxone administration (21, 22). Courtney et al. (2023)

found that lack of knowledge about opioid overdose and naloxone administration among laypersons were among the major barriers for using naloxone at overdose scenes (21).

Despite the importance of reducing rates of opioid-involved overdoses, no prior studies have specifically assessed opioid overdose knowledge and perceived naloxone competency among Black urban residents in Indiana. In order to develop effective interventions, it is necessary to better understand the individual- and community-level factors associated with opioid overdose knowledge and perceived naloxone competency. By leveraging recent probability samples across multiple Indiana cities, we aim to provide baseline data critical for informing community-based interventions designed to better ensure that all communities have access to effective means of reducing opioid-involved overdose fatality rates.

Accordingly, the objectives of this study were (1) to measure the level of knowledge and perceived competency regarding opioid overdose and naloxone administration in recent probability samples of Black urban communities in Indiana, (2) investigate racial and other sociodemographic correlates of knowledge and perceived competency, and (3) assess possible relationships, if any, between community-level factors (e.g., proportion of Black residents in the community, community-level income and employment rates) and knowledge and perceived competency.

# Methods

## Ethics statement

This study was approved by the Indiana University Institutional Review Board (IUIRB), which determined that the research met the federal and university criteria for exemption (approved protocol ID: 16953). Informed consent was obtained for all participants. Waiver of documentation of consent was approved by the IUIRB.

## Sample and administration

This study was part of the Multi-Sector and Multi-Level Community-Driven Approaches to Remove Structural Racism and Overdose Deaths in Black Indianapolis Communities (MACRO-B), a project that includes opioid overdose and naloxone administration community education programming to reduce opioid overdose deaths in Black Indianapolis communities. As part of the project, data were collected from March 2023 to May 2023 via mail and web-based household probability community surveys with samples of non-institutionalized adults aged 18 years or older. Demographic characteristics of survey respondents are shown in Table S1. Data collection was managed by the Indiana University Center for Survey Research. Probability sampling was used to select respondents representative of eight urban zip code areas in Indiana with Black populations greater than 40%, including inner-city Indianapolis (46202, 46205, 46208, and 46218) and northern Indiana communities—Gary (46408), Merrillville (46410), South Bend (46628), and Fort Wayne (46806). The target intervention communities in Indianapolis were selected based on the high percentage of Black residents and high rates of opioid overdose events and deaths. The comparison communities in northern Indiana were selected by zip-code-level matching on the proportion of Black population, percentages of families living in poverty, population size, and the proportion of children eligible for free/reduced lunch, using the data from the United States Census Bureau and Indiana State Department of Health overdose surveillance reports (7, 19). The intervention and comparison communities were similar in terms of the four matching variables while they were geographically distant (i.e., 125 to 150 miles away).

The study adhered to the guidelines established by the American Association for Public Opinion Research (AAPOR) (23). Survey administration proceeded in sequential stages. On day

1, households were mailed a preliminary letter containing a $1 pre-incentive and instructions for completing an online survey, along with notification that a $20 gift card would be provided upon completion. Reminder letters were sent to non-respondents on day 10 and day 20, with the second reminder including a paper version of the survey and a self-addressed, stamped return envelope. A third reminder with a paper version of the survey was mailed on day 35, followed by a final reminder on days 50-55, encouraging participation either online or by mail. The overall response rate was 23%, calculated using AAPOR Response Rate Formula #4 (23). A detailed summary of the survey administration timeline is available in Figure S2.

# Measures

## Knowledge on opioid overdose and naloxone administration

To assess knowledge, 10 items assessing opioid overdose knowledge were adapted from the Opioid Overdose Knowledge Scale (OOKS) (24). In the current sample, the internal consistency reliability of the 10-item knowledge scale was modest (Cronbach's alpha = 0.41). This is consistent with prior research suggesting that lower Cronbach's alpha values are acceptable for knowledge-based assessments, particularly when measuring diverse domains of factual information (25, 26). The questionnaire included 6 true/false items and 4 multiple-choice items designed to test basic knowledge about identifying the risks, signs, and symptoms of opioid overdose, as well as understanding the proper use of naloxone (see Table S3 for these items). Each correct answer earned one point, resulting in a possible score range of 0 to 10 for knowledge on opioid overdose and naloxone administration. We calculated mean scores by race, ensuring that all scores were weighted accordingly.

## Perceived competency to manage opioid overdoses

To assess the perceived level of competency in managing opioid overdoses, we asked the survey respondents to rate their agreement with 7 items on a Likert-type scale ranging from 1 to 5 (see Table S3 for these items). These items were also derived from the Opioid Overdose Knowledge Scale (OOKS) and the Opioid Overdose Attitudes Scale (OOAS) (24). Each item was scored individually, and appropriate reverse coding was made so that a higher score always indicated a higher perceived level of competency (Cronbach's alpha = 0.87). We then calculated the weighted mean scores of all 7 items by race.

### Individual-level and zip code-level predictors

One of the key variables in this study was race. In analyses, variable responses were dummy coded as White, Black, and Other. Due to the small sample sizes for specific groups (Asian = 22, American Indian or Alaska Native = 21, and Native Hawaiian or Pacific Islander = 6), we combined those individuals with the "Other" category to ensure sufficient numbers for meaningful analyses (27). Geographic area was determined by the eight zip code areas, which were then dummy coded by region as inner-city Indianapolis (46202, 46205, 46208, and 46218) and other urban areas such as Gary (46408), Merrillville (46410), South Bend (46628), and Fort Wayne (46806). Several demographic factors were included as covariates: age group (18-24, 25-34, 35-44, 45-54, 55-64, 65-74, and 75+), biological sex at birth (women, men), ethnicity (Non-Latino, Latino), educational level (some college or higher, high school/GED or less), and household income, which had the following categories: less than $15,000; $15,000–$24,999; $25,000–$34,999; $35,000–$49,999; $50,000–$74,999; $75,000–$99,999; and $100,000 or more. For analysis purposes, income was collapsed into three broader categories (over $100,000, $35,000-$99,999, less than $35,000). Other covariates included duration of residence in the community (more than 3 years, 1 to 3 years, less than a year) and history with opioid overdose

(yes/no). The variable for opioid overdose history was defined by whether individual had personally experienced an opioid overdose or had a family member or close friend who died from an opioid overdose. Additionally, zip code-level factors were included such as proportion of Black residents, number of individuals with income below the 200 percent of federal poverty level (FPL<200%), median household income, proportion of individuals with high school or higher educational attainment among those who were 25 years or older, and employment rate. These zip-code level data are shown in Table S4. These factors were obtained from the 2022 American Community Survey 5-year estimates (7).

## Statistical analysis

Out of the 772 survey respondents, 47 cases with missing information were excluded using listwise deletion, resulting in a final analytic sample of 725 respondents. Given the low rate of missingness 6% (28), listwise deletion was appropriate to maintain a consistent analytic sample across variables (29). Descriptive statistics included unweighted frequencies and weighted proportions for demographic characteristics and correct responses on opioid overdose and naloxone administration questions. Weighted mean scores were calculated for perceived competency items. Categorical variables were compared across racial groups using adjusted chi-square tests with design-based Rao-Scott corrections. Continuous variables were compared using design-based F-tests, accounting for the complex survey design.

Multilevel analysis models were then constructed to identify factors associated with two outcomes: (a) knowledge and (b) perceived competency regarding opioid overdose and naloxone administration. Following Lee et al. (2015), nested models were sequentially built, beginning with a null model, then adding individual-level socioeconomic variables, overdose experience,

zip code-level predictors, and cross-level interactions (30). All analyses were conducted using R version 4.3.2 (31).

# Results

## Demographic characteristics

Table 1 provides a breakdown of the demographic and socioeconomic characteristics of the survey respondents categorized by race, including White (n = 359), Black (n = 347), and Other racial groups (n = 60). There were significant differences by race observed for the following variables: ethnicity ($p = 0.002$), age group ($p < 0.001$), educational attainment ($p = 0.001$), household income ($p < 0.001$), and opioid overdose history ($p = 0.003$). Region, sex, and length of time lived in the communities were not significantly different by race.

<< Insert Table 1 about here >>

## Weighted scores for knowledge on opioid overdose and naloxone administration and perceived competency level to manage opioid overdoses by race

Table 2 presents the unweighted frequencies and weighted proportions of responses to the 10 questions on opioid overdose and naloxone administration knowledge, along with the weighted mean scores of 7 items that assess perceived competency in managing opioid overdose. The average score on the 10 questions assessing knowledge on opioid overdose and naloxone administration showed significant differences by race ($p < .001$). White participants had the highest scores (Mean = 6.65, *SD* = 1.86), followed by other racial groups (Mean = 5.75, *SD* = 1.89), and then Black participants (Mean = 5.70, *SD* = 1.79). In contrast, perceived competency to manage opioid overdose, assessed through 7 survey items, did not show significant variation

across racial groups ($p$ = 0.478). White participants had a mean score of 2.95 ($SD$ = 1.05), Black participants scored 2.82 ($SD$ = 0.92), and Other racial groups scored 2.96 ($SD$ = 1.07).

Some individual knowledge questions (components of the overall knowledge score) showed significant variation by racial group, whereas none of the individual weighted mean scores for perceived competence items (components of the overall weighted mean competency score) indicated significant differences among racial groups.

<< Insert Table 2 about here >>

## Factors associated with knowledge on opioid overdose and naloxone administration scores

Table 3 presents the multilevel analysis of factors influencing knowledge about opioid overdose and naloxone administration. The model included both individual and zip code-level predictors, as well as interaction terms. In the model, Black (β = -1.74, $p$ = 0.005) participants had significantly lower opioid overdose and naloxone administration knowledge scores compared to their White counterparts. Education level also played a significant role, with participants having a high school education or less scoring lower than those with some college or higher education (β = -0.31, $p$ = 0.044). Income was another significant predictor; compared to those earning > \$100,000, those earning < \$100,000 showed lower knowledge scores (β = -0.47, $p$ = 0.009 for those earning \$35,000 – \$99,999 and β = -0.66, $p$ = 0.002 for those earning less than \$35,000). Notably, participants with a history of opioid overdose demonstrated higher knowledge scores (β = 0.83, $p$ = <0.001). For the length of time lived in the community, individuals who lived in their area for one to three years scored lower than those who lived there for more than three years (β = -0.42, $p$ = 0.012). Zip code-level predictors showed no significant relationship with knowledge scores. However, a significant cross-level interaction was found

between Black race and FPL<200%, indicating that Black residents living in poorer neighborhoods displayed lower knowledge scores than White counterparts (β = 1.06, $p$ = 0.039). The intraclass correlation coefficient (ICC) for the null model was 0.028, indicating that 2.8% of the total variance in the knowledge level was due to differences between the zip codes.

<< Insert Table 3 about here >>

## Factors associated with perceived competency on opioid overdose and naloxone administration

Table 4 summarizes the multilevel model analysis on perceived competency in managing opioid overdose and administering naloxone. At the individual level, race was not a significant predictor, but age was. The youngest age group (18-24 years) reported significantly lower perceived competency (β = -0.34, $p$ = 0.013) compared to the 35-44 age group, as did the oldest groups, aged 65-74 (β = -0.45, $p$ = 0.001) and 75+ (β = -0.33, $p$ = 0.013). No significant differences were noted for the other age groups. The only other significant predictor of perceived competency was having a personal or familial history of opioid overdose, which was associated with a higher score (β = 0.61, $p$ < 0.001). The ICC of the null model was 0.004, indicating that only 0.4% of the variance in perceived competency was due to differences between zip codes. Random effects analysis showed minimal variability across zip codes.

<< Insert Table 4 about here >>

# Discussion

This study is one of the first to examine knowledge and perceived competency regarding opioid overdose and naloxone administration among racially diverse urban residents in Indiana. Although previous research has documented rising overdose deaths among Black Americans

nationally, little was known about how knowledge and readiness to respond to overdoses vary by race and neighborhood conditions at the local level. By using probability samples across major urban areas of Indiana, our study provides novel baseline data that highlights significant differences. White urban residents scored the highest on knowledge items, while Black residents scored the lowest. Furthermore, these differences in level of knowledge were associated with community-level poverty via an interaction effect, with Black residents living in high-poverty neighborhoods showing the lowest scores. These findings reveal important structural and contextual barriers to overdose prevention, and suggest that future overdose prevention efforts that are specifically tailored by race and socioeconomic status may help optimize prevention of fatal opioid-involved overdoses.

Our finding that Black urban residents scored lower on knowledge questions around opioid overdose and naloxone administration compared to White urban residents is consistent with prior research (32, 33). Likewise, the relatively low knowledge scores across all urban Indiana residents in the sample aligns with previous research indicating that despite naloxone's relatively widespread availability, knowledge about naloxone is lacking (22, 34-36). This could likely be improved through peer-led educational initiatives (37-41), which are successful in improving participants' knowledge about opioid overdose and naloxone administration.

Seo et al. (2023) found that Black residents in Indianapolis felt they lacked sufficient educational opportunities regarding opioid overdose and naloxone usage compared to White urban residents (42). However, they expressed a readiness to learn about and carry naloxone for overdose response, particularly when the education and support came from trusted figures within their community such as Black church leaders (42). Addressing the gap observed in this study might be achievable by implementing culturally tailored education programs (43, 44) involving

community leaders who grasp the cultural nuances and can communicate effectively with Black residents about the importance of naloxone administration (45).

Also consistent with previous studies (46, 47), our findings indicated that having a history of opioid overdose, whether personal or through the death of a family member or close friend, was associated with significantly higher knowledge scores. This suggests that firsthand experience or proximity to opioid-related events may serve as a catalyst for increased awareness and understanding (46, 47), potentially due to greater engagement with medical professionals or support services following such events. Interestingly, no significant associations were found between zip code-level predictors and knowledge scores except the poverty level of the community. Research has shown that poverty and socioeconomic status are linked to educational access and health literacy (48) thereby affecting health outcomes (49). Additionally, aligning with previous studies (47, 50) several socioedemographic factors were significantly related with knowledge scores. Men, individuals with lower household income and educational attainment, and those in lower-income brackets exhibited lower knowledge scores. Strategies such as targeted education, improved access to resources, local partnerships, tailored messaging, financial incentives, and ongoing evaluation, may help address knowledge disparities among men, lower-income, and less-educated groups, though exploring their effectiveness lies beyond the scope of this study. Additionally, we found a cross-level interaction between race and poverty level. This interaction emphasizes the need for interventions that address both race and socioeconomic status such that more Black residents in high-poverty areas are provided with tailored educational opportunities on opioid overdose and naloxone administration.

Regarding perceived competency in managing opioid overdose and naloxone administration, age emerged as a significant predictor, with both the youngest (18-24 years) and

oldest age groups (65-74 and 75 years or older) reporting lower perceived competency. This may indicate a lack of practical experience or exposure to training opportunities among these cohorts. Notably, having an opioid overdose history significantly enhanced perceived competency, aligning with our previous findings regarding knowledge scores. This relationship suggests that experiential learning may play an important role in developing confidence in overdose management. In a study based in Nebraska, Cooper-Ohm et al. (2023) found an insignificant relationship between past history and familiarity with naloxone in cities of Omaha and Lincoln, however a significant relationship was found in regions outside these two cities (51). Furthermore, the ICC results from the null models in this study provide insights into the variability of knowledge and perceived competency related to opioid overdose management and naloxone administration across different urban communities. Specifically, the ICC showed that 2.8% of the variance in knowledge was due to geographical differences. Urban communities with limited access to resources could benefit from educational campaigns and support services that address their specific needs (52). By acknowledging the community context, public health practitioners could develop more effective strategies to share important information about opioid overdose prevention and naloxone use (37, 53, 54). On the other hand, the ICC for perceived competency was only 0.4%, which indicates that differences in perceived competency across zip codes are minimal, regardless of the availability of educational resources in these communities.

The findings of this study should be cautiously interpreted within the context of its limitations. By focusing on specific zip codes and recruiting survey respondents from only those zip codes, there may be limited generalizability, potentially applying only to the settings with similar characteristics and environments. Nonetheless, the chosen zip codes were deliberately selected from urban areas with higher proportions of Black residents and elevated rates of

overdose events and deaths compared to other areas in Indiana, rendering the findings of our study meaningful. Additionally, since the survey relied on self-reported data, it is susceptible to response bias and recall bias. Furthermore, we could not assess temporal relationships due to the cross-sectional nature of the survey. Also, the responses to opioid overdose items may have been subject to social desirability bias, potentially leading to overestimation of perceived competence. While our survey included 10 knowledge items and 7 competency items, these items may not have fully captured the breadth of understanding and skills needed for effective overdose response. Finally, the lack of qualitative data limits our ability to explore the underlying reasons behind observed disparities, such as structural barriers or cultural factors affecting knowledge and competency. Future research should incorporate qualitative approaches to better capture the nuances of opioid overdose knowledge and response competency.

This study aimed to assess the level of knowledge and perceived competency regarding opioid overdose and naloxone administration among urban Indiana residents and to examine racial and community-level differences. Our findings demonstrate that significant gaps in knowledge exist, particularly among Black residents living in high-poverty areas, underscoring the value of tailored education initiatives. While strategies to reduce opioid-involved deaths are multifaceted, ensuring layperson access to naloxone, improving knowledge of overdose recognition and response, and strengthening naloxone administration self-efficacy are critical components. As part of the MACRO-B project, we are currently providing community-based overdose prevention education, naloxone administration training, and naloxone distribution. Moving forward, we plan to conduct longitudinal and qualitative research to better understand the mechanisms underlying these differences and to evaluate the long-term effectiveness and sustainability of our intervention efforts.

# Acknowledgements

This work was supported by the Indiana University Center for Survey Research (CSR). We thank the CSR for assisting in administering community surveys in our target study area which greatly assisted the research although they may not agree with all of the interpretations/conclusions of this paper.

# Funding statement

This project was supported by the Office of Minority Health (OMH) of the U.S. Department of Health and Human Services (HHS) as part of a financial assistance award (CPIMP221346). The contents are those of the authors and do not necessarily represent the official views of, nor an endorsement, by OMH/OASH/HHS, or the U.S. Government. For more information, please visit https://www.minorityhealth.hhs.gov/. There was no additional external funding received for this study.

# Captions for supporting information files

S1 Table. Demographic characteristics of survey respondents: March-May 2023 (N = 772)

S2 Fig. Survey administration timeline and follow-up procedure

S3 Table. Weighted frequencies and percentages for knowledge on opioid overdose and naloxone administration by race: March-May, 2023 (N = 772)

S4 Table. Zip-code level predictors: 2022 American Community Survey 5-year estimates

# References


1. Carter L, McGinty, A., Sloan, L., Malcezewski, M., & Sandusky, G. Manner of Death and Fentanyl Related Drug Overdose Trends in Marion County, Indiana, US from 2018 to 2021. Forensic Medicine and Anatomy Research. 2023;11(2):30-7.
2. (NCHS). NCHS. National Center for Health Statistics (NCHS). Drug Overdose Death in the United States 2002-2022. 2024.
3. (KFF) KFF. Opioid overdose deaths and opioid overdose deaths as a percent of all drug overdose deaths 2023 [Available from: https://www.kff.org/other/state-indicator/opioid-overdose-deaths/.
4. Gibbons JB, Harris SJ, Solomon KT, Sugarman O, Hardy C, Saloner B. Increasing overdose deaths among Black Americans: a review of the literature. Lancet Psychiatry. 2023;10(9):719-26.
5. Gondre-Lewis MC, Abijo T, Gondre-Lewis TA. The Opioid Epidemic: a Crisis Disproportionately Impacting Black Americans and Urban Communities. J Racial Ethn Health Disparities. 2023;10(4):2039-53.
6. Kyei EF, Leveille S. Opioid Misuse and Opioid Overdose Mortality Among the Black Population in the United States: An Integrative Review. Policy Polit Nurs Pract. 2023;24(3):208-18.
7. Bureau UC. American Community Survey (ACS). 2024.
8. (FSSA) IFSSA. Analysis of opioid overdose mortality and vulnerability index in Indiana. 2023.
9. (SAMHSA) SAaMHSA. SAMHSA Opioid Overdose Prevention TOOLKIT. 2018.
10. (NIH). NIoH. Naloxone DrugFacts. National Institute on Drug Abuse (NIDA). 2022.
11. Bennett AS, Bell A, Doe-Simkins M, Elliott L, Pouget E, Davis C. From Peers to Lay Bystanders: Findings from a Decade of Naloxone Distribution in Pittsburgh, PA. J Psychoactive Drugs. 2018;50(3):240-6.
12. Razaghizad A, Windle SB, Filion KB, Gore G, Kudrina I, Paraskevopoulos E, et al. The Effect of Overdose Education and Naloxone Distribution: An Umbrella Review of Systematic Reviews. Am J Public Health. 2021;111(8):1516-7.
13. Lambdin BH, Zibbell J, Wheeler E, Kral AH. Identifying gaps in the implementation of naloxone programs for laypersons in the United States. Int J Drug Policy. 2018;52:52-5.
14. Dayton L, Tobin K, Falade-Nwulia O, Davey-Rothwell M, Al-Tayyib A, Saleem H, et al. Racial Disparities in Overdose Prevention among People Who Inject Drugs. J Urban Health. 2020;97(6):823-30.
15. Takemoto E, Bolton A, Goetz CT. Inequities in naloxone administration among fatal overdose decedents by race and ethnicity in Pennsylvania, 2019-21. Addiction. 2024;119(8):1400-9.
16. Ray B, Lowder E, Bailey K, Huynh P, Benton R, Watson D. Racial differences in overdose events and polydrug detection in Indianapolis, Indiana. Drug Alcohol Depend. 2020;206:107658.
17. Smart R, Pardo B, Davis CS. Systematic review of the emerging literature on the effectiveness of naloxone access laws in the United States. Addiction. 2021;116(1):6-17.
18. (FDA) USFDA. FDA Approves First Over-the-Counter Naloxone Nasal Spray 2023.
19. Recovery N. Progress Report. The Indiana State Government. 2023.

20. Bennett AS, Freeman R, Des Jarlais DC, Aronson ID. Reasons People Who Use Opioids Do Not Accept or Carry No-Cost Naloxone: Qualitative Interview Study. JMIR Form Res. 2020;4(12):e22411.
21. Courtney J, Ferrarotti, A., Tran, T., & Titus-Lay, E. Layperson Knowledge and Perceived Barriers With Obtaining Naloxone. Journal of Contemporary Pharmacy Practice. 2023;70(3):16-26.
22. Schlosser A, Habecker P, Bevins R. Harm reduction in the Heartland: public knowledge and beliefs about naloxone in Nebraska, USA. Harm Reduct J. 2022;19(1):22.
23. (AAPOR). TAAfPOR. Standard Definitions: Final Dispositions of Case Codes and Outcome Rates for Surveys. 10th 2023 [Available from: https://aapor.org/wp-content/uploads/2023/05/Standards-Definitions-10th-edition.pdf.
24. Williams AV, Strang J, Marsden J. Development of Opioid Overdose Knowledge (OOKS) and Attitudes (OOAS) Scales for take-home naloxone training evaluation. Drug Alcohol Depend. 2013;132(1-2):383-6.
25. Streiner DL. tarting at the beginning: An introduction to coefficient alpha and internal consistency. Journal of Personality Assessment. 2003;80(1):99-103.
26. KS T. The Use of Cronbach's Alpha When Developing and Reporting Research Instruments in Science Education. Research in Science Education. 2018;48:1279-96.
27. Flanagin A, Frey T, Christiansen SL, Committee AMAMoS. Updated Guidance on the Reporting of Race and Ethnicity in Medical and Science Journals. JAMA. 2021;326(7):621-7.
28. Dong Y, Peng CY. Principled missing data methods for researchers. Springerplus. 2013;2(1):222.
29. Roderick J. A. Little DBR. Statistical Analysis with Missing Data: John Wiley & Sons, Inc.; 2002.
30. Lee CG, Seo DC, Middlestadt SE, Lin HC. Does the Relationship Between Cigarette Smoking and Other Key Health Behaviors Vary by Geographic Area Among US Young Adults? A Multilevel Analysis. Int J Behav Med. 2015;22(4):481-8.
31. Team RC. R: A Language and Environment for Statistical Computing. R Foundation for Statistical Computing, 2023 [Available from: https://www.R-project.org/.
32. Barboza GE, Angulski K. A descriptive study of racial and ethnic differences of drug overdoses and naloxone administration in Pennsylvania. Int J Drug Policy. 2020;78:102718.
33. Khan MR, Hoff L, Elliott L, Scheidell JD, Pamplin JR, 2nd, Townsend TN, et al. Racial/ethnic disparities in opioid overdose prevention: comparison of the naloxone care cascade in White, Latinx, and Black people who use opioids in New York City. Harm Reduct J. 2023;20(1):24.
34. Bessen S, Metcalf SA, Saunders EC, Moore SK, Meier A, McLeman B, et al. Barriers to naloxone use and acceptance among opioid users, first responders, and emergency department providers in New Hampshire, USA. Int J Drug Policy. 2019;74:144-51.
35. Urmanche AA, Harocopos A. Experiences Administering Naloxone Among People in Different Social Roles: People Who Use Opioids and Family Members and Friends. J Drug Issues. 2023;53(3):475-89.
36. Hohmann LA, Krauss Z, Patel J, Marley GT. Public Perceptions of Community Pharmacy-Based Naloxone Services: A National Cross-Sectional Survey. Pharmacy (Basel). 2022;10(6).

37. Enich M, Flumo R, Campos S, Flores N, Sullivan N, Mellor J, et al. Overdose education and naloxone distribution program design informed by people who use drugs and naloxone distributors. Prev Med Rep. 2023;35:102374.
38. Dos Santos B, Farzan Nipun, R., Maria Subic, A., Kubica, A., Rondinelli, N., Marentette, D., ... & M Orkin, A. Virtual and remote opioid poisoning education and naloxone distribution programs: a scoping review. medRxiv. 2023:11.
39. Owczarzak J, Weicker N, Urquhart G, Morris M, Park JN, Sherman SG. "We know the streets:" race, place, and the politics of harm reduction. Health Place. 2020;64:102376.
40. Hanson BL, Porter RR, Zold AL, Terhorst-Miller H. Preventing opioid overdose with peer-administered naloxone: findings from a rural state. Harm Reduct J. 2020;17(1):4.
41. Neale J, Brown C, Campbell ANC, Jones JD, Metz VE, Strang J, et al. How competent are people who use opioids at responding to overdoses? Qualitative analyses of actions and decisions taken during overdose emergencies. Addiction. 2019;114(4):708-18.
42. Seo DC, Satterfield N, Alba-Lopez L, Lee SH, Crabtree C, Cochran N. "That's why we're speaking up today": exploring barriers to overdose fatality prevention in Indianapolis' Black community with semi-structured interviews. Harm Reduct J. 2023;20(1):159.
43. (SAMHSA) SAaMHSA. Substance Abuse and Mental Health Services Administration: The Opioid Crisis and the Black/African

American Population: An Urgent Issue. . 2020.
44. Hughes M, Suhail-Sindhu S, Namirembe S, Jordan A, Medlock M, Tookes HE, et al. The Crucial Role of Black, Latinx, and Indigenous Leadership in Harm Reduction and Addiction Treatment. Am J Public Health. 2022;112(S2):S136-S9.
45. Winhusen T, Brown, P. L., Ernst, T. I., Ingram, T. I., & Tyehimba, K. A. Increasing naloxone distribution in Black Communities: A case study. . Journal of the National Medical Association. 2024;116(4):424.
46. Buresh M, Gicquelais RE, Astemborski J, Kirk GD, Mehta SH, Genberg BL. Fatal overdose prevention and experience with naloxone: A cross-sectional study from a community-based cohort of people who inject drugs in Baltimore, Maryland. PLoS One. 2020;15(3):e0230127.
47. Resko SM, Pasman E, Hicks DL, Lee G, Ellis JD, O'Shay S, et al. Naloxone Knowledge and Attitudes Towards Overdose Response Among Family Members of People who Misuse Opioids. J Community Health. 2024;49(1):70-7.
48. Raghupathi V, Raghupathi W. The influence of education on health: an empirical assessment of OECD countries for the period 1995-2015. Arch Public Health. 2020;78:20.
49. Schillinger D. Social Determinants, Health Literacy, and Disparities: Intersections and Controversies. Health Lit Res Pract. 2021;5(3):e234-e43.
50. Lazar M, Davenport L. Barriers to Health Care Access for Low Income Families: A Review of Literature. J Community Health Nurs. 2018;35(1):28-37.
51. Cooper-Ohm S, Habecker P, Humeniuk R, Bevins RA. Factors Associated with Gaps in Naloxone Knowledge: Evidence from a 2022 Great Plains Survey. Res Sq. 2023.
52. Rose J, Arikat L, Gusoff G, Pollack CE. Mechanisms to Improve Health Through Community Land Trusts. J Urban Health. 2023;100(2):389-97.
53. Nolen S, Trinidad AJ, Jordan AE, Green TC, Jalali A, Murphy SM, et al. Racial/Ethnic differences in receipt of naloxone distributed by opioid overdose prevention programs in New York City. Res Sq. 2023.

54. Knudsen HK, Walker DM, Mack N, Kinnard EN, Huerta TR, Glasgow L, et al. Reducing perceived barriers to scaling up overdose education and naloxone distribution and medications for opioid use disorder in the United States in the HEALing (Helping End Addiction Long-Term(R)) communities study. Prev Med. 2024;185:108034.

Table 1. Demographic Characteristics by Race: March-May 2023, (N = 772)

| | White (n = 359) | Black (n = 347) | Other (n = 60) | |
|---|---|---|---|---|
| Variable | n (%) | n (%) | n (%) | *p*-value |
| Ethnicity | | | | 0.002 |
| Non-Latine | 323 (87.5) | 330 (95.6) | 43 (76.1) | |
| Latine | 35 (12.5) | 13 (4.4) | 16 (23.9) | |
| Age group | | | | <0.001 |
| 18-24 years | 38 (14.7) | 27 (15.2) | 11 (22.9) | |
| 25-34 years | 82 (30.3) | 36 (9.6) | 10 (16.0) | |
| 35-44 years | 63 (16.6) | 54 (14.4) | 16 (31.8) | |
| 45-54 years | 39 (8.9) | 54 (16.6) | 9 (10.0) | |
| 55-64 years | 47 (12.3) | 67 (22.3) | 6 (6.2) | |
| 65-74 years | 56 (10.6) | 71 (14.7) | 5 (9.6) | |
| 75 years or older | 33 (6.6) | 38 (7.2) | 2 (3.5) | |
| Biological sex at birth | | | | 0.600 |
| Women | 217 (55.0) | 247 (59.2) | 41 (63.7) | |
| Men | 142 (45.0) | 99 (40.8) | 19 (36.3) | |
| Educational attainment | | | | 0.001 |
| Some college or higher | 288 (67.0) | 250 (47.3) | 48 (73.5) | |
| High school /GED or less | 71 (33.0) | 97 (52.7) | 12 (26.5) | |
| Household income | | | | <0.001 |
| Upper, more than $100,000 | 94 (29.8) | 46 (11.5) | 14 (27.3) | |
| Middle, $35,000 - $99,999 | 161 (46.2) | 151 (33.8) | 21 (33.3) | |
| Lower, less than $35,000 | 99 (29.8) | 148 (54.7) | 23 (39.4) | |
| Length of time lived in the communities | | | | 0.554 |
| More than three years | 244 (65.6) | 261 (72.7) | 41 (70.1) | |
| One year to three years | 83 (27.3) | 66 (20.1) | 15 (25.7) | |
| Less than a year | 32 (7.1) | 19 (7.2) | 4 (4.2) | |
| Opioid overdose history[a] | | | | 0.003 |
| No | 264 (69.1) | 275 (78.4) | 53 (93.7) | |
| Yes | 95 (30.9) | 72 (21.6) | 6 (6.3) | |
| Region | | | | 0.996 |
| Indianapolis | 204 (54.3) | 175 (54.8) | 40 (54.8) | |
| Other | 155 (45.7) | 172 (45.2) | 20 (45.2) | |

*Notes.* GED = General Educational Development. Frequencies are unweighted, and proportions are weighted. Frequencies may not sum to the total due to missing observations. Differences between groups were examined via weighted adjusted Chi-Square tests.

[a] Opioid overdose history include individuals with personal opioid overdose experience as well as experiences involving the death of a family member or close friend due to opioid overdose.

Table 2. Weighted Scores for Knowledge on Opioid Overdose and Naloxone Administration and Perceived Competency Level to Manage Opioid Overdoses by Race: March-May, 2023 (N = 772)

| | White (n = 359) | Black (n = 347) | Other (n = 60) | |
|---|---|---|---|---|
| | | Mean (SD) | | *p*-value[a] |
| *Knowledge on Opioid Overdose and Naloxone Administration (10 Questions)* | 6.65 (1.86) | 5.70 (1.79) | 5.75 (1.89) | <0.001 |
| *Perceived Competency to Manage Opioid Overdose (7 items)* | 2.95 (1.05) | 2.82 (0.92) | 2.96 (1.07) | 0.478 |

*Notes.* Frequencies may not sum to the total due to missing observations.
[a] Corresponding p-values were calculated via weighted design-based F-tests.

Table 3. Multilevel Model Analysis of Knowledge on Opioid Overdose and Naloxone Administration (N=723)

| Variable | β | SE | *p*-value |
|---|---|---|---|
| **Fixed Effects** | | | |
| *Individual-level predictors* | | | |
| Intercept | | | |
| Race | | | |
| White | Ref | | |
| Black | **-1.74**** | 0.62 | 0.005 |
| Other | -1.02 | 0.89 | 0.252 |
| Ethnicity | | | |
| Non-Latino | Ref | | |
| Latino | -0.40 | 0.24 | 0.102 |
| Age group | | | |
| 18-24 years | 0.39 | 0.24 | 0.109 |
| 25-34 years | 0.38 | 0.22 | 0.086 |
| 35-44 years | Ref | | |
| 45-54 years | -0.01 | 0.24 | 0.964 |
| 55-64 years | 0.11 | 0.24 | 0.634 |
| 65-74 years | 0.26 | 0.25 | 0.309 |
| 75 years or older | -0.07 | 0.31 | 0.813 |
| Biological sex at birth | | | |
| Women | Ref | | |
| Men | **-0.36**** | 0.13 | 0.007 |
| Educational attainment | | | |
| Some college or higher | Ref | | |
| High school /GED or less | **-0.31*** | 0.16 | 0.044 |
| Household income | | | |
| Upper, more than $100,000 | Ref | | |
| Middle, $35,000 - $99,999 | **-0.47**** | 0.18 | 0.009 |
| Lower, less than $35,000 | **-0.66**** | 0.21 | 0.002 |
| Length of time lived in the communities | | | |
| More than three years | Ref | | |
| One year to three years | **-0.42*** | 0.17 | 0.012 |
| Less than a year | -0.37 | 0.29 | 0.206 |
| Opioid overdose history[a] | | | |
| Yes | **0.83***** | 0.16 | <0.001 |
| No | Ref | | |
| Region | | | |
| Indianapolis | 0.33 | 0.26 | 0.206 |
| Other | Ref | | |
| *Zip code-level predictors* | | | |
| Black proportion | -1.04 | 1.43 | 0.467 |
| FPL < 200%[c] | -0.26 | 0.71 | 0.715 |

| | | | |
|---|---|---|---|
| *Cross-level interaction* | | | |
| Race (Black) * FPL < 200% | **1.06**[*] | 0.51 | 0.039 |

| **Random Effects** | | 95% CI[b] |
|---|---|---|
| $\tau_{00}$ (Level-2) | 0.26 | 0.01 – 0.35 |
| $\sigma^2$ (Level-1) | 1.70 | 1.60 – 1.77 |

*Note.* Ref = reference group. GED = General Educational Development. FPL = Federal Poverty Level. Due to missing data on the variables, the analytical sample was reduced to 723.

[a] Opioid overdose history include individuals with personal opioid overdose experience as well as experiences involving the death of a family member or close friend due to opioid overdose.

[b] 95% profile confidence interval

[c] The number of individuals for FPL<200% was divided by 10,000 to reduce its scale in analysis.

[*] $p < .05$ [**] $p < .01$ [***] $p < .001$.

Table 4. Multilevel Model Analysis of Perceived Competency on Opioid Overdose and Naloxone Administration (N=723)

| Variable | β | SE | *p*-value |
|---|---|---|---|
| **Fixed Effects** | | | |
| *Individual-level predictors* | | | |
| Intercept | | | |
| Race | | | |
| White | Ref | | |
| Black | -0.58 | 0.29 | 0.093 |
| Other[a] | 0.63 | 0.49 | 0.203 |
| Ethnicity | | | |
| Non-Latine | Ref | | |
| Latine | -0.25 | 0.13 | 0.068 |
| Age group | | | |
| 18-24 years | **-0.34**[*] | 0.13 | 0.013 |
| 25-34 years | -0.11 | 0.12 | 0.392 |
| 35-44 years | Ref | | |
| 45-54 years | -0.15 | 0.14 | 0.264 |
| 55-64 years | -0.04 | 0.13 | 0.764 |
| 65-74 years | **-0.45**[**] | 0.14 | 0.001 |
| 75 years or older | **-0.33**[*] | 0.17 | 0.013 |
| Biological sex at birth | | | |
| Women | Ref | | |
| Men | 0.05 | 0.07 | 0.480 |
| Educational attainment | | | |
| Some college or higher | Ref | | |
| High school /GED or less | -0.06 | 0.09 | 0.456 |
| Household income | | | |
| Upper, more than $100,000 | Ref | | |
| Middle, $35,000 - $99,999 | -0.08 | 0.10 | 0.429 |
| Lower, less than $35,000 | -0.08 | 0.11 | 0.469 |
| Length of time lived in the communities | | | |
| More than three years | Ref | | |
| One year to three years | -0.02 | 0.09 | 0.817 |
| Less than a year | -0.09 | 0.16 | 0.571 |
| Opioid overdose history[b] | | | |
| Yes | **0.61**[***] | 0.09 | <0.001 |
| No | Ref | | |
| Region | | | |
| Indianapolis | -0.07 | 0.10 | 0.491 |
| Other | Ref | | |
| *Zip code-level predictors* | | | |
| Black proportion | 0.24 | 0.55 | 0.664 |
| FPL < 200%[c] | -0.08 | 0.31 | 0.878 |

| | | | |
|---|---|---|---|
| *Cross-level interaction* | | | |
| Race (Black) * FPL < 200% | 0.41 | 0.28 | 0.144 |

| **Random Effects** | | 95% CI[b] |
|---|---|---|
| $\tau_{00}$ (Level-2) | 0.00 | 0.00 – 0.00 |
| $\sigma^2$ (Level-1) | 0.90 | 0.89 – 0.99 |

*Note.* Ref = reference group. GED = General Educational Development. FPL = Federal Poverty Level. Due to missing data on the variables, the analytical sample was reduced to 723.

[a] Opioid overdose history include individuals with personal opioid overdose experience as well as experiences involving the death of a family member or close friend due to opioid overdose.

[b] 95% profile confidence interval

[c] The number of individuals for FPL<200% was divided by 10,000 to reduce its scale in analysis.

$^{*}p < .05$ $^{**}p < .01$ $^{***}p < .001$

**Revisions in Response to Reviewer Comments on "Opioid Overdose and Naloxone Administration Knowledge and Perceived Competency in A Probability Sample of Indiana Urban Communities with Large Black Populations" (Manuscript # PONE-D-24-50788)**

## Editor:

1. Please ensure that your manuscript meets PLOS ONE's style requirements, including those for file naming. The PLOS ONE style templates can be found here:

- We have edited to follow the style requirements.

2. Please provide an amended statement that declares *all* the funding or sources of support (whether external or internal to your organization) received during this study, as detailed online in our guide for authors at http://journals.plos.org/plosone/s/submit-now. Please also include the statement "There was no additional external funding received for this study." in your updated Funding Statement. Please include your amended Funding Statement within your cover letter. We will change the online submission form on your behalf.

- Done as requested.

3. We note that you have indicated that there are restrictions to data sharing for this study. For studies involving human research participant data or other sensitive data, we encourage authors to share de-identified or anonymized data. However, when data cannot be publicly shared for ethical reasons, we allow authors to make their data sets available upon request. For information on unacceptable data access restrictions, please see http://journals.plos.org/plosone/s/data-availability#loc-unacceptable-data-access-restrictions. Before we proceed with your manuscript, please address the following prompts: If there are ethical or legal restrictions on sharing a de-identified data set, please explain them in detail (e.g., data contain potentially identifying or sensitive patient information, data are owned by a third-party organization, etc.) and who has imposed them (e.g., a Research Ethics Committee or Institutional Review Board, etc.). Please also provide contact information for a data access committee, ethics committee, or other institutional body to which data requests may be sent. Please update your Data Availability statement in the submission form accordingly.

- We will make datasets available upon request. We have updated Data Availability Statement as requested.

4. Please remove any funding-related text from the manuscript and let us know how you would like to update your Funding Statement.

- In response to Request #2 above, we have already amended our Funding Statement and included it within our revised cover letter. We have removed all funding-related text from the abstract and main narrative.

5. Please include captions for your Supporting Information files at the end of your manuscript, and update any in-text citations to match accordingly. Please see our Supporting Information guidelines for more information: http://journals.plos.org/plosone/s/supporting-information.

- Done as requested.

## Reviewer #1:

The response rate is relatively low 23%

- While the overall response rate of 23% is relatively low, it is consistent with the rates typically reported in contemporary household-based probability surveys using mail and web modes without in-person follow-up. Recent reports from the American Association for Public Opinion Research (AAPOR) attribute declining response rates to broader trends such as survey fatigue and reduced willingness to participate. To minimize potential nonresponse bias, we employed probability sampling, survey weighting, and multiple follow-up strategies, including reminder letters, mailed paper surveys, and incentives. Furthermore, the demographic composition of our final analytic sample closely matched census benchmarks for the target communities, suggesting limited risk of nonresponse bias.
- This is also supported by the findings from a replicated methodological study by Keeter and his colleagues (Keeter et al., 2006) which compared standard recruitment (25% response rate) with a rigorous recruitment (50% response rate) using random-digit-dial (RDD) methods. Across 77 of 84 variables, the surveys produced statistically indistinguishable results, indicating that unit nonresponse within this response rate range did not meaningfully compromise data quality (Keeter et al., 2006). The study also found that even respondents hardest to reach or those who partially completed interviews did not differ systematically in key behaviors or attitudes (Keeter et al., 2006) - further supporting the robustness of survey estimates despite modest response rates. [Keeter, S., Kennedy, C., Dimock, M., Best, J., & Craighill, P. (2006). Gauging the impact of growing nonresponse on estimates from a national RDD telephone survey. *International Journal of Public Opinion Quarterly*, *70*(5), 759-779]

What are the steps taken to calculate demographic matching?

- We used four zip-code level demographic matching variables: Black racial proportion, families living in poverty, population size, and proportion of children eligible for free/reduced lunch. Detailed information has been provided in text (Lines 144 – 152).

You used OOKS and OOAS without any clarifications for the psychometric validation?

- We apologize for the confusion. While OOKS (on knowledge) and OOAS (on attitudes) are companion scales, we only used OOKS to measure knowledge. As requested, we have computed and reported the psychometric properties of both the knowledge scale and the perceived level of competency in managing opioid overdoses scale in our sample. The Cronbach's alpha for the perceived level of competency was 0.87 but it was 0.41 for the knowledge scale. Although the Cronbach's alpha in our sample was modest for the knowledge scale, we have provided justification for its acceptability by citing two supporting articles. Specifically, one should not expect a knowledge test to function in a similar psychometric manner to a scale intended to measure a singular construct, since not all knowledge items measure the same constructs. We have revised the text accordingly (Lines 165 – 170).

There is a significant finding that Black participants in high-poverty areas had lower knowledge scores than their White counterparts. However, the mechanisms or contributing factors are not well explored.

- We appreciate the comments. We agree that the mechanisms underlying the observed differences are important to explore. However, as this study represents baseline, exploratory findings within a larger intervention project, no prior analyses among Indiana urban residents were available to further investigate potential contributing factors. We have clarified this in the revised manuscript and highlighted that while the results suggest important differences, identifying specific mechanisms will require further longitudinal and qualitative research (Lines 365 – 368).

In limitation section you should mention social bias and lack of qualitative data and using only 10 knowledge and 7 competency items

- As requested, we have added the limitation of potential social desirability bias and the limited number of knowledge and competency items, and the absence of qualitative data (Lines 361 – 368).

The method section should include response rate the different approach

- As requested, we have revised the Methods section to explicitly report the overall response rate (23%) and added a detailed description of the sequential survey administration approach, including mailings, reminders, and incentive use (Lines 153 – 162). To further clarify the data collection process, we have also created and included a flowchart (Figure S2) that visually summarizes the survey administration timeline and participation outcomes.

Table 2 is dense

- As requested, we have revised Table 2 to streamline its content and improve readability. Additionally, we have moved specific survey items to Table S4 to make the main table more concise while still providing detailed information for readers who wish to review it.

Add some diagrams or graphs

- Done as suggested. As mentioned above, we have developed a flowchart illustrating the sample selection and survey administration process to enhance clarity. The flowchart has been added as Figure S2 in the revised manuscript.

Language and grammar editing

- Done as suggested.

## Reviewer #2:

In abstract should be explained briefly related to IMRAD, furthermore in abstract also explained what becomes Novelty.

- As requested, we have revised the Abstract to follow the IMRAD structure more clearly, providing distinct sections for the background, methods, results, and conclusions. In addition, we have added a sentence highlighting the novelty of the study, specifically noting how our findings contribute to new evidence regarding the interaction between structural factors (e.g., community poverty) and race in shaping opioid overdose knowledge (Lines 42 – 62).

In the introduction, I have not found any Research Gap, so researchers need to improve; where from the Research Gap, researchers can give something new from this research, and I have not found what is offered by researchers as Novelty. Literature review explains how previous studies work, and you can tell what you find in previous research and what you find new from this research.

- ➢ As requested, we have revised the Introduction and literature review section to clarify the research gap and the novelty of the study (Lines 84 – 86 & 111 – 118).

In the methodology may need to be explained in more detail related to the research subject so that it becomes clear who is the subject.

- ➢ Done as suggested (Lines 144 – 152)

In results and discussion should give an idea of the basis of the research's purpose and discuss in-depth the findings in the field, and from there, researchers can give something new from this research.

- ➢ As requested, we have revised the opening section of the Discussion section to better connect our findings back to the study's research purpose, emphasize the novelty of our work, and more clearly explain how our findings contribute new insights to the field (Lines 286 – 298).

Please add Conclusions must certainly be able to answer from the purpose of this study.

- ➢ Done as suggested (Lines 369 – 380)

## Reviewer #3:

Thank you for providing me the opportunity to review this manuscript. Overall I found this manuscript to be an interesting read, describing the disparity in knowledge of naloxone and opioid overdoses and be impacted by systemic issues related to race. It is a timely reminder that we all need to do more in our work to ensure the right messages are reaching the right people, regardless of race or other factors.

- ➢ Thank you for your thoughtful and encouraging feedback.

I think there are two areas of improvement needed for this manuscript: 1) being concise, and 2) clearly describing all statistical analyses completed.

- ➢ In view of the reviewer comments, we have revised the manuscript to be more concise and have clarified the description of all statistical analyses.

I am a firm believer of using less words where possible, and I would suggest the authors look at areas where they could reduce the word count. At times, some sections felt overly described. Perhaps the section that stood out to me the most was when describing listwise deletion and why it was used. I would assume most people reading would understand listwise deletion, and that it's use when describing could be said it in fewer words. As another suggestion to shorten the methods, is provide the survey instrument as a supplementary to reduce the word count in the methods when describing the survey. I appreciate the authors have submitted to a non-addiction or drug and alcohol journal, and therefore at times need to describe certain concepts in greater details.

- As requested, we have revised the manuscript to make it concise where possible, including the explanation of listwise deletion. Additionally, we have moved the detailed description of the survey procedure to Figure S2, as suggested.

I was confused as the methods made no mention of using chi-square or t-test analyses, and yet table 1 and table 2 have results from these tests. It was not until i noticed the footnotes of each table that i realized these tests were completed for each table. It would be important to ensure this is adequately described within the statistical analyses section. Did the authors utilize a Bonferroni correction for the chi-square test? Are there further analyses completed that as a reader I am not aware of?

- We apologize for the lack of clarity in the Methods section. We have revised the Statistical Analyses subsection to explicitly state that we conducted adjusted chi-square tests with design-based Rao-Scott corrections for categorical variables and design-based F-tests for continuous variables in Tables 1 and 2 (Lines 214 – 217). No Bonferroni correction was applied for these descriptive comparisons for two reasons. First, even with Bonferroni adjustment with 10 pairwise comparisons (using 0.005 rather than 0.05 as the p value threshold), all the reported statistical significance stays unchanged. Second, these analyses were intended to characterize the (sub)samples rather than hypotheses-driven statistical tests.

## Reviewer #4:

The abstract is intelligible and accurately describes the objective and results obtained. It is a sufficient summary of the contents of the paper. In the abstract, the results should be explored. The CONCLUSIONS could be directly related to the results found in the study.

- Thank you for the thoughtful feedback. As suggested, we have revised the abstract to explain the results and to ensure the conclusions are directly tied to the findings of the study.

METHODS: The methods were described and organized in sufficient detail. However, the chapter is quite long. I would suggest to the authors to summarize it. A suggestion would be to present another flowchart with the methodological organization.

- As suggested, we have summarized the methods section to improve clarity and conciseness and have included an additional flowchart in Figure S2.

RESULTS: The results are interesting. The authors clearly highlighted the information collected using the methods described to meet the study objectives. The tables with the presentation of the results are organized and clear. The authors identify the limitations of the study in the end of the manuscript, but it is necessary to indicate suggestions for future research.

- As suggested, we have provided suggestions for future research (Lines 378 – 380).

CONCLUSION. The authors could present a clear and objective conclusion.

- As suggested, we have revised the Conclusion section to present a clearer and more objective summary of the findings (Lines 369 – 380).

REFERENCES: The authors cite appropriate and actual papers for comments/discussion made

- Thank you.
