# Supplementary material for: Opioid overdose and naloxone administration knowledge and perceived competency in a probability sample of Indiana urban communities with large Black populations
Source: PLoS One. 2025 Jul 15;20(7):e0328444. doi: 10.1371/journal.pone.0328444 (PMC12262839; doi:10.1371/journal.pone.0328444)
Supplement: Attachment — Submitted filename: response to reviewers_PLoS One_0628.docx [file pone.0328444.s010.docx]

**Revisions in Response to Reviewer Comments on “Opioid Overdose and Naloxone Administration Knowledge and Perceived Competency in A Probability Sample of Indiana Urban Communities with Large Black Populations” (Manuscript #** **PONE-D-24-50788R1)**

**Reviewer #1:**

1. Section 2.2, paragraph 1.explain how these areas were chosen beyond demographic criteria

- Revised to include additional details on area selection (Lines 146-157, 160-161).

2. Section 2.3.3. ...defined by whether individual had personally experienced an opioid overdose or had a family member or close friend who died

- Revised to clarify the construction of the opioid overdose history variable. Specifically, the binary variable was based on responses to two survey items: (1) whether the respondent had personally experienced an opioid overdose and (2) whether a family member or close friend had died from an opioid overdose (Lines 208-212).

3. Section 2.3.3, Table 1. Confusing

- Revised the table notes to clarify the interpretation of numbers and how the opioid overdose history variable was constructed (Lines 559-565).

4. Tables 3 and 4, Results narrative - Results sections rely only on tables

- Expanded the results narrative to integrate key findings from tables (Lines 263-265, 282-283, 296-298).

5. Discussion paragraphs 1 and 2 closely mirror Results

- Revised the discussion to reduce overlap and enrich interpretations (Lines 303-305, 310-312, 325-328).

6. Abstract is dense and underrepresents key findings in plain language.

- Revised for clarity and a better representation of main findings (Lines 42-50, 60-63).

7. Citations 5, 7, 40 used heavily

- Citations 5 and 40 are foundational but are now balanced with additional supporting references.

8. No discussion of studies that show minimal or no racial difference in naloxone administration

- Addressed with literature and integrated into the discussion (Lines 318-322)

9. No figures

- A figure in the supplementary document has been created using PACE digital diagnostic tool and uploaded.

10. Table captions could better explain

- Revised as suggested (Lines 559-565).

**Reviewer #2:**

1. This article may be corrected as follows:
   Please look again at the author's guidelines on abstract, where abstract is mapped with background, objective, result and conclusion. A good abstract already looks what is novelty in this article.

- Revised to align with the structured format and to emphasize novelty of the study.

2. In the introduction, I have not found any Research Gap, so researchers need to improve; wherefrom the Research Gap, researchers can give something new from this research, and I have found what is offered by researchers as Novelty. In results and discussion should give an idea of the basis of the research's purpose and discuss in-depth the findings in the field, and from there, researchers can give something new from this research.

- Revised the introduction to clarify the research gap and articulate the study’s novelty (Lines 112-122). The discussion has also been expanded to better connect with the study aims (Lines 303-305, 310-312, 318-322, 325-328).

**Reviewer #3:**

1. The authors have addressed comments by reviewers sufficiently and I have no further suggestions for this manuscript.

- Thank you.

**Reviewer #4:** 
The revisions that you made to the manuscript are very effective in addressing the remaining concerns. Following the review of the previous version, considering the scientific merit, the originality of the manuscript and the reading of the new version, the recommendation is: "ACCEPT". Congratulation one your fine research!

- Thank you.
